# Integrated microbial–metabolomic analysis reveals how fermentation contributes to the unique flavor of African Arabica coffee

**DOI:** 10.1016/j.fochms.2025.100344

**Published:** 2025-12-18

**Authors:** Gilberto Vinícius de Melo Pereira, Alexander da Silva Vale, Ana Isabel Ribeiro-Barros, Luiz Roberto Saldanha Rodrigues, Gisela Manuela de França Bettencourt Mirção, Bernadete Camilo, Inocência da Piedade Ernesto Tapaça, Vitoria de Mello Sampaio, Satinder kaur Brar, Carlos Ricardo Soccol

**Affiliations:** a Federal University of Paraná (UFPR), Department of Bioprocess Engineering and Biotechnology, Curitiba, PR, Brazil; b Forest Research Center, Associate Laboratory TERRA, School of Agriculture, University of Lisbon, Tapada da Ajuda, 1349-017 Lisbon, Portugal; c Federal University of Technology Paraná (UTFPR), Department of Chemistry and Biology, Curitiba, PR 80230-901, Brazil; d Higher Polytechnic Institute of Manica (ISPM), Division of Agriculture, Biotechnology Program, Manica, Mozambique; e Department of Civil Engineering, Lassonde School of Engineering, York University, North York, Toronto, ON M3J 1P3, Canada

**Keywords:** Arabica coffee, Coffee fermentation, Volatile organic compounds, Metagenomics, Flavor development

## Abstract

Post-harvest fermentation is a decisive stage in shaping the flavor complexity of Arabica coffee. In this study, we mapped for the first time the microbial-driven flavor metabolic network underlying the fermentation of high-quality African coffee, using a combined metabolomic, meta-barcoding, and metagenomic approach applied to samples from Chimanimani National Park, Mozambique. Over 72 h of spontaneous fermentation, chemical analyses revealed rapid sucrose hydrolysis, lactic acid accumulation, and the formation of 74 volatile compounds. These transformations were driven by a previously unreported core microbiome (*Leuconostoc–Hanseniaspora–Galactomyces* axis), whose functional repertoire (1791 genes) highlighted the Ehrlich pathway and ester biosynthesis as central flavor routes. Among the volatiles formed, linalool, phenylethyl alcohol, and ethyl acetate were most abundant, emerging as predictive drivers of the floral and fruity notes identified in the resulting high-quality coffee beverage (score 87.25 ± 0.25). This study underscores microbial terroir as a key factor adding value to emerging African origins.

## Highlights

• First multi-omics study of coffee fermentation in Africa.
• Core microbiome (*Leuconostoc*, *Hanseniaspora*, *Galactomyces*) not previously reported.
• Shotgun metagenomics revealed pathways driving key flavor compounds.
• Linalool, phenylethyl alcohol, and ethyl acetate drove major flavor formation.
• A high-quality coffee was produced with fruity–floral notes linked to fermentation metabolites.

## 1 Introduction

Arabica coffee (*Coffea arabica* L.) is the predominant species in the global specialty coffee industry. Genomic evidence shows that *C. arabica* is an allotetraploid that arose via natural hybridization between *C. canephora* and *C. eugenioides* roughly 350,000–610,000 years ago, a pivotal event that shaped its genetic architecture and long-term evolutionary trajectory (Salojärvi et al., 2024). Domestication began in the highlands of south-western Ethiopia, later expanding to Yemen in the 15th–16th centuries; from there, coffee spread to India, Southeast Asia, and the Americas, establishing the Typica/Bourbon lineages that enabled adaptation across diverse agroecological zones and the emergence of distinct regional sensory profiles (Montagnon et al., 2021). These trajectories, combined with variety, altitude, climate, and soil, drive terroir-linked differences in flavor and aroma (de Junqueira et al., 2019; Wiele et al., 2025).

Along with origin-related factors, post-harvest fermentation is now recognized as a critical phase that defines coffee's chemical and sensory identity (de Pereira et al., 2024). During wet processing, microbial succession typically begins with yeasts and lactic acid bacteria (LAB), followed by acetic acid bacteria (AAB) as pH, oxygen availability, and substrate pools shift. This ecological progression drives targeted biotransformations (e.g., sugar and amino-acid catabolism) that generate flavor precursors and aroma-active volatiles (de Pereira et al., 2019; dos Gomes et al., 2024). In biochemical terms, yeast-mediated Ehrlich-type pathways and esterification yield higher alcohols and esters, while LAB/AAB metabolism produces organic acids that modulate acidity and redox, accelerating mucilage breakdown and reshaping the beans' chemical matrix (de Pereira et al., 2019). Across origins (Australia, Brazil, Colombia, Honduras, Ecuador, and Thailand), multi-omics surveys routinely report more than 500 taxa on average in coffee fermentations and correlate microbial diversity with metabolite profiles and cup attributes, underscoring microbial succession as a central determinant of quality (Cruz-O'Byrne et al., 2021; da Vale et al., 2021; Elhalis, Cox, & Zhao, 2020; Palumbo et al., 2024; Zhang et al., 2019).

Beyond ecological and genetic importance, African coffees are frequently described as having bright acidity with floral and fruity character. These attributes are linked to monoterpenes (e.g., linalool, geraniol, limonene) and related aroma-actives that survive roasting and/or are released from glycosidic precursors, imparting jasmine, citrus, and berry-like notes (Zakidou et al., 2021). Together, these data support a chemistry-based rationale for regional flavor archetypes in specialty coffee (Ogutu et al., 2022; Zakidou et al., 2021). In contrast, many Central/South American coffees are typified by nutty, cocoa, and caramel-like profiles dominated by pyrazines and furans generated during Maillard/roasting chemistry, contributing “roasty,” nutty, and sweet impressions (Cascos et al., 2024; Viljoen, 2019).

Within southeastern Africa, Mozambique has recently emerged as a promising origin through agroforestry initiatives in Gorongosa and Chimanimani, where shade, altitude, and biodiversity conservation align with improved physical/chemical bean attributes and specialty potential (Cassamo et al., 2023; da Tapaça et al., 2024). Despite Africa being the genetic birthplace of *Coffea arabica*, how microbial succession during post-harvest fermentation links to metabolite pathways and to the chemical and sensory quality of African coffees remains unknown. This gap underscores the importance of exploring microbial terroir, which is the connection between local microbial communities and sensory expression, as a framework for understanding the unique flavors of African coffees.

In this study, we investigate post-harvest fermentation of washed Arabica produced in Chimanimani, Mozambique, integrating metabolomics, amplicon (metabarcoding), and shotgun metagenomics to (i) resolve microbial succession, (ii) map flavor-associated metabolites (with emphasis on alcohols/esters, organic acids, and terpenoid-derived volatiles), and (iii) link these biochemical pathways to cup-relevant aroma. By providing high-resolution evidence of how microbial dynamics drive chemical transformations and generate aroma-active metabolites, this work advances the mechanistic understanding of microbial terroir in African coffees and clarifies how native microbiota shape Arabica chemistry and sensory quality at its center of origin.

## 2 Materials and methods

### 2.1 Study area and fermentation conditions

The study was conducted in Monte Tsetsera, Chimanimani National Park (CNP) (between latitude 19.37833° South, and longitude 32.80944° East), a conservation area located in Manica Province, Mozambique. CNP is classified as a key biodiversity area of global relevance (KBA) and exhibits unique environmental and ecological characteristics that are increasingly being explored for Arabica coffee cultivation, with new plantations currently under development (Timberlake et al., 2020). Fermentation trials were carried out in June 2024 during the coffee harvest season. Approximately 12 kg of ripe *Coffea arabica* cherries were manually harvested and depulped. The resulting mucilage-coated beans were evenly divided into three aliquots of approximately 4  kg each. Each aliquot was transferred into a sterile plastic container containing 4 L of fresh, non-chlorinated water to initiate spontaneous fermentation under on-farm conditions. Fermentation was conducted over 72 h at ambient temperature, which ranged from 20 to 30 °C during the day and 14–20 °C at night. The beans were manually stirred every 24 h, and samples of the fermentation liquid fraction were collected at 0, 24, 36, 48, 62, and 72 h. The samples were immediately frozen at −20 °C for subsequent molecular and physicochemical analyses. After fermentation, the beans were thoroughly washed to remove residual mucilage, following the fully washed processing method. The washed beans were placed in sieves and dried in an oven with air circulation at 35 ± 3 °C until reaching a moisture content of approximately 12 %. Finally, the dried beans were vacuum-packed in plastic bags and stored at −20 °C until further analysis.

### 2.2 Dynamics of sugars and organic acids during coffee fermentation

The major primary metabolites, including sugars (sucrose, glucose, fructose) and organic acids (lactic, acetic, malic, succinic, citric, and propionic), were quantified in samples collected throughout the fermentation process. For this analysis, 1 mL aliquots were centrifuged at 10,000 ×*g* for 10 min and subsequently filtered through a 0.22 μm filter.

For sugar analysis, a Metrohm 850 Professional IC chromatograph was used, equipped with pulsed amperometric detection (PAD) with a gold electrode and an autosampler model 919. A Carbopac PA100 pre-column (4 × 50 mm) and a Carbopac PA100 column (4 × 250 mm) were employed for compound separation. Instrument control and data processing were performed using the MagIC 3.2 software. The sample injection volume was 20 μL, and the mobile phase consisted of 91 mM sodium hydroxide at a flow rate of 0.7 mL/min, with the column maintained at 30 °C. The amperometric detector with a gold electrode was set to 35 °C, operating with a pulse sequence of 300 ms at 0.05 V, 50 ms at 0.55 V, and 200 ms at −0.1 V.

Organic acids were quantified using an HPLC system (Agilent Technologies, Waldbronn, Germany) equipped with a refractive index detector (RID). Compound separation was performed using a Hiplex-H column (300 × 7.7 mm) (Bio-Rad, Richmond, CA, USA) under isocratic conditions. The mobile phase consisted of 5.0 mM H₂SO₄ at a flow rate of 0.6 mL/min for 30 min. The column temperature was maintained at 60 °C, while the RID detector operated at 50 °C.

### 2.3 Dynamics of volatile organic compounds during coffee fermentation

The identification of volatile organic compounds (VOCs) generated during fermentation was performed using gas chromatography coupled with mass spectrometry (GC–MS). For the analysis, 3 mL of the liquid fraction from the fermentation was transferred to 20 mL vials and hermetically sealed. The extraction of volatiles was carried out by solid-phase microextraction (SPME) using a DVB/CAR/PDMS fiber (Supelco Co., Bellefonte, PA, USA), which was exposed for 30 min at 60 °C. Subsequently, the compounds adsorbed on the fiber were thermally desorbed at 260 °C and directly introduced into the chromatographic system. The separation of the compounds was performed using a gas chromatograph equipped with an SH-Rtx-5MS capillary column (30 m × 0.25 mm × 0.25 μm). The GC operating conditions were set as follows: column oven temperature set at 60 °C, injection temperature at 260 °C, and detector maintained at 250 °C. Helium was used as the carrier gas, with a flow rate of 1 mL/min, a column pressure of 57.4 kPa, and a split ratio of 1:20. Mass spectrometry was conducted within the range of 30–250 *m*/*z*, with the ion source maintained at 250 °C. The identification of volatile compounds was performed by comparing the obtained mass spectra with reference libraries or spectra of standard compounds. For quantification, phenylethyl alcohol was selected as the external standard because it was one of the most abundant and consistently detected VOCs in the chromatographic runs and exhibited highly stable ionization behavior under the GC–MS conditions employed. Standard solutions ranging from 5 to 100 μmol L^−1^ were prepared to construct calibration curves, which showed excellent linearity and reproducibility across the analytical series. Given the structural diversity of the 74 VOCs identified and the unavailability of analytical standards for all compounds, volatile concentrations were expressed as μmol L^−1^ of headspace as phenylethyl-alcohol equivalents, following established semi-quantitative procedures for VOC analysis in coffee fermentation (de Pereira et al., 2016).

### 2.4 Dynamics of microbial communities during coffee fermentation by metagenetic profiling

The DNA from samples collected throughout the fermentation process was extracted to characterize microbial succession. An aliquot of 5 mL was first centrifuged at 500×*g* (4 °C, 5 min) to remove large particulate matter. The supernatant was then centrifuged at 4000×*g* (4 °C, 15 min), and the resulting microbial pellet was washed with phosphate-buffered saline (PBS). Genomic DNA was extracted using the PowerSoil DNA Isolation Kit (Qiagen, Carlsbad, CA, USA), following the manufacturer's protocol. DNA quality and quantity were assessed using a Nanodrop spectrophotometer and visualized on a 0.8 % agarose gel electrophoresis. To investigate the microbial ecology and metabolic potential during coffee fermentation, two complementary strategies were adopted for molecular analysis: amplicon-based metagenetic profiling and shotgun metagenomics.

Amplicon-based metagenetic profiling was then performed to assess microbial succession across fermentation stages (0, 24, 36, 48, 62, and 72 h). The V3–V4 hypervariable region of the 16S rRNA gene was amplified for bacterial profiling using primers 341F (CCTACGGGNGGCWGCAG) and 805R (GACTACHVGGGTATCTAATCC), and the ITS region for fungi 1737F (GGAAGTAAAAGTCGTAACAAGG) and 2043R (GCTGCGTTCTTCATCGATGC) (Wiele et al., 2025).Paired-end sequencing (2 × 150 bp) was performed on the Illumina NextSeq platform. Sequence quality control, filtering, and Amplicon Sequence Variant (ASV) inference were performed using QIIME2 v.2023.7 with DADA2. Taxonomic classification was carried out using Naïve Bayes classifiers trained on the SILVA v.138 database (bacteria) and the UNITE database (fungi). Microbial diversity was evaluated using alpha diversity indices (Shannon, Chao1, Simpson), and beta diversity patterns were analyzed via PCA.

### 2.5 Shotgun metagenomic sequencing

Shotgun sequencing was used to characterize the functional gene repertoire at the final stage of fermentation (72 h). This specific time point was selected because it represented the most stable and differentiated phase of the microbial succession, as confirmed by the plateauing of alpha diversity indices and the clear clustering observed in PCA analyses. At 72 h, dominant microbial taxa had already been established, enabling a more accurate and representative recovery of metagenome-assembled genomes (MAGs).

One nanogram of DNA was fragmented and tagged using the NexteraXT library preparation kit (Illumina). Library size and quality were assessed using GelBot (Loccus), and sequencing was conducted using paired-end reads (2 × 150 bp) on an Illumina NextSeq system. Sequence quality was analyzed using Trimmomatic v.0.39, removing low-quality regions and adapter sequences (Bolger et al., 2014). Metagenome assembly was performed with SPAdes v.3.15.4 using the -meta parameter (Bankevich et al., 2012). Functional and taxonomic annotation of the assembled genomes was carried out using Prokka v.1.14.6 (Seemann, 2014), while the taxonomic classification of the reads was performed with the Kraken2 classifier, using the NCBI RefSeq database (Wood & Salzberg, 2014). Only sequences related to fungi and bacteria were considered in the analysis, as they represent the main organisms involved in microbial interaction during coffee bean fermentation. From the assembled metagenome, binning was performed using MaxBin2.0, which clusters metagenomic contigs into genome bins representing distinct microbial organisms. The phylogenetic classification of each bin was determined using GTDB-Tk v2, which assigns taxonomic classifications based on the Genome Taxonomy Database.

### 2.6 Functional annotation and mapping of metabolic pathways

Functional annotations were conducted on sequences obtained from shotgun sequencing to characterize identified genes. Initially, gene annotation based on the Kyoto Encyclopedia of Genes and Genomes (KEGG) was performed using the KAAS tool with default parameters in Single Best Hit mode (http://www.genome.jp/kegg/kaas/). Following the KEGG orthology assignment, genes were mapped onto metabolic pathways using the iPath platform (http://pathways.embl.de).

Subsequently, translated gene sequences were aligned against the eggNOG database using the BLASTP algorithm (WU-BLAST 2.0; http://blast.wustl.edu). As described by Xie et al., 2013), a single gene can be assigned to multiple functional categories within the eggNOG system, providing a comprehensive analysis of genomic functions. This analysis enabled the identification and classification of genes encoding different families of carbohydrate-active enzymes, including glycoside hydrolases, glycosyltransferases, and polysaccharide lyases.

### 2.7 Reconstruction of metagenome-assembled genome for *Leuconostoc*-driven metabolic routes

To investigate the unexpected persistence of *Leuconostoc* throughout the fermentation process, metagenome-assembled genomes (MAGs) were reconstructed using shotgun metagenomic data obtained at the final fermentation stage (72 h). Quality-filtered reads were assembled using SPAdes v.3.15.4 with the “--meta” parameter, and contigs were binned with MaxBin2.0. MAG quality was assessed using CheckM v.1.2.2, and only bins with ≥80 % completeness and ≤ 10 % contamination were retained. Taxonomic assignment of these high-quality MAGs was performed using GTDB-Tk v.2.0, resulting in the identification of four genomes corresponding to *Leuconostoc citreum*, *Lactococcus cremoris*, *Weissella confusa*, and *Lactococcus lactis* subsp. *lactis*. Functional annotation of the predicted genes was conducted using Prokka v.1.14.6 for gene prediction and feature identification, followed by KO assignment via the KEGG Automatic Annotation Server (KAAS) and additional functional classification using eggNOG-mapper v.2.

### 2.8 Chemical profiling of fermented coffee beans

The fermented coffee beans were ground, and the particle size was standardized using a 35-mesh sieve. For the analysis of non-volatile compounds (sugars and organic acids), 500 mg of the sample was transferred to a flask containing 20 mL of ultrapure water and subjected to extraction in a shaking water bath at 60 °C for 90 min. After extraction, the suspensions were centrifuged at 10.000×*g* for 10 min, and the supernatants were filtered through a 0.22 μm membrane filter. The quantification of sugars and organic acids was performed by HPLC under the same conditions described above.

The ground coffee samples were also used for the analysis of volatile compounds. For this, 3 g of the sample was placed in a 20 mL vial, and the extraction was performed using solid-phase microextraction (SPME) with a DVB/CAR/PDMS fiber, which was exposed for 30 min at 60 °C. The adsorbed compounds were then thermally desorbed at 260 °C and injected directly into the gas chromatography system coupled with mass spectrometry (GC–MS). The chromatographic separation and detection conditions followed the previously described methodology.

### 2.9 Sensory analysis

The green coffee beans were classified according to the parameters established by the Specialty Coffee Association (SCA) (sca.coffee/research/coffee-standards). The samples exhibited a greenish-blue colour, a moisture content of 11.7 %, and a water activity of 0.54. A 100 g sample from screen size 16 and above was roasted using a Probat Leogap TP 2 sample roaster. The roasting protocol included a charge temperature of 136.1 °C, a total roasting time of 9 min and 41 s, and a development phase of 85 s (14.6 %) after the first crack. The roasting curve was monitored and recorded using Cropster software (Fig. S1). After roasting, the sample underwent an 18-h resting period before being evaluated in a blind cupping session by three Q Graders, following the SCA sensory evaluation methodology (https://sca.coffee/research/coffee-standards). Tastify software was used to compile the average attribute descriptions of the sample.

### 2.10 Statistical analysis

Statistical significance was determined using a post-hoc comparison of means with Tukey's test. The analyses were performed using SAS software, version 7.0 (Statistical Analysis System, Cary, NC, USA). A two-tailed *p*-value <0.05 was considered statistically significant.

## 3 Results and discussion

### 3.1 Chemical transformations during coffee fermentation

#### 3.1.1 Sugars

**Fig. 1:**
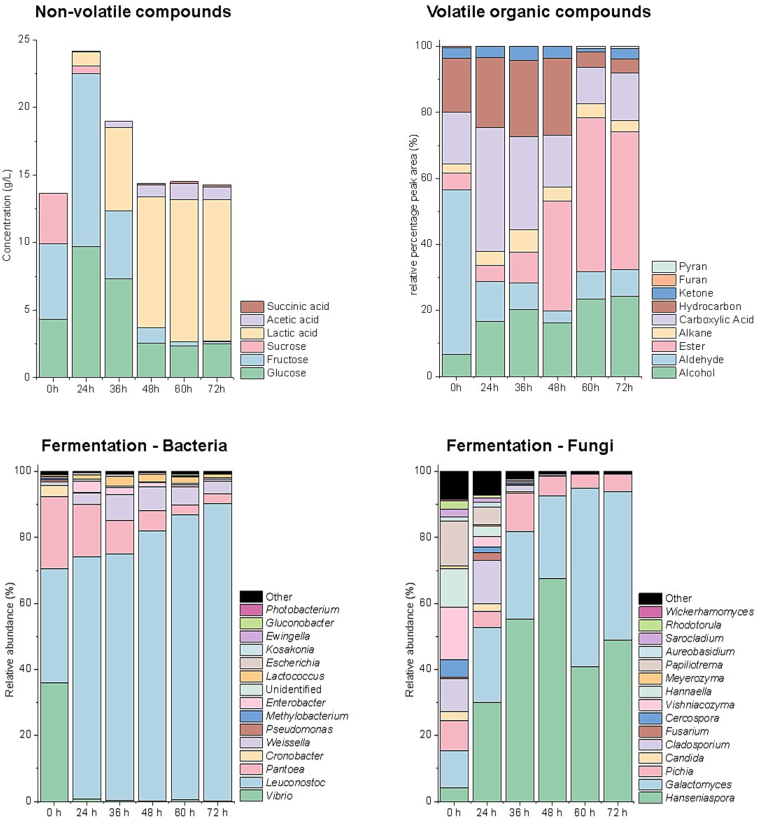
Metabolic and microbial profile of the liquid fraction during spontaneous fermentation of coffee beans carried out in Chimanimani National Park, Mozambique. Metabolic characterization includes the concentration of non-volatile compounds (glucose, fructose, sucrose, lactic acid, acetic acid, and succinic acid) and the relative distribution of chemical groups of volatile compounds (alcohols, aldehydes, esters, alkanes, fatty acids, hydrocarbons, ketones, furans, and pyranos).Bacterial and fungal genera with relative abundance ≥0.2 % over time are presented.

The initial composition of coffee pulp showed high levels of fructose (5.57 ± 0.71 g/L), glucose (4.34 ± 0.60 g/L), and sucrose (3.72 ± 0.33 g/L) (Fig. 1), with sucrose rapidly hydrolyzed during the first hours of fermentation by microbial invertases and glycosidases, releasing additional monosaccharides (Braga et al., 2023). As fermentation progressed, both monosaccharides were gradually consumed by fermentative microorganisms. By the end of the process, only residual levels of glucose (2.48 ± 0.26 g/L) and fructose (0.19 ± 0.01 g/L) remained, indicating active microbial metabolism and efficient sugar utilization. In most coffee fermentations, glucose is preferentially consumed over fructose, leading to higher residual fructose at the end of the process (da Vale et al., 2019; de Carvalho Neto et al., 2018). However, in this study, fructose depletion exceeded glucose, suggesting an inversion of the typical sugar utilization pattern. This unusual behavior coincided with the predominance of *Hanseniaspora* (Fig. 1), a genus frequently linked to a fructophilic phenotype, where strains preferentially metabolize fructose even in the presence of glucose (Cabral et al., 2015; Ciani & Fatichenti, 1999). The underlying mechanism involves high-affinity transport systems for fructose combined with weaker glucose repression, enabling more efficient assimilation of fructose under mixed sugar conditions. Importantly, fructose metabolism by *Hanseniaspora* has been associated with the enhanced production of esters and higher alcohols that impart fruity and floral sensory attributes (Valera et al., 2024), indicating that this altered sugar assimilation pathway may directly contribute to the distinctive flavor profile of the resulting coffee beverage.

#### 3.1.2 Primary and secondary metabolites

The fermentation process induced significant changes in both primary (non-volatile) and secondary (volatile) metabolites. Among the organic acids, lactic acid showed the most pronounced increase, reaching 10.47 ± 0.90 g/L at 72 h (Fig. 1). Its accumulation is consistent with the dominance of LAB, which is typically associated with carbohydrate metabolism, pectin degradation, and microbial restructuring in coffee fermentations (de Pereira et al., 2020). In parallel, moderate increases in acetic and malic acids were observed, likely linked to the combined metabolic activity of yeasts, LAB, and a limited population of AAB present during the fermentation.

Seventy-four volatile organic compounds (VOCs) were identified throughout the fermentation process, grouped into alcohols (15), aldehydes (15), alkanes (10), carboxylic acids (7), esters (9), hydrocarbons (10), ketones (6), furans (1), and pyrans (1) (Fig. 1 and Table 1).PCA revealed two distinct clusters corresponding to early (0, 24, and 36 h) and late (48, 60, and 72 h) fermentation stages, indicating a metabolic shift from coffee pulp precursors to microbial-derived metabolites such as alcohols and esters (Fig. S2).

**Table 1:** Concentration of volatile compounds (μmol L^−1^) identified in the liquid fraction during fermentation of Mozambican coffee beans.

Compounds	Chemical group	Time (h)					
		0	24	36	48	60	72
1-Butanol, 3-methyl	*Alcohol*	0	0	0	0	36.51	40.07
1-Hexanol		50.51	54.22	22.70	74.90	87.38	61.58
(+−)-5-Methyl-2-hexanol		0	62.22	0	0	0	0
2-Heptanol		39.70	0	22.74	19.25	161.36	29.37
1-Octen-3-ol		0	19.97	36.57	38.03	43.61	44.09
3-Octanol		0	0	0	0	20.60	19.89
3-Ethyl-4-methylpentan-1-ol		0	35.22	45.11	36.87	35.69	0
1-Hexanol, 2-ethyl-		48.88	36.81	33.79	31.65	30.59	19.48
1-Octanol		0	45.75	30.51	29.49	26.96	18.93
Linalool		20.62	90.96	205.90	314.83	333.03	475.45
Phenylethyl Alcohol		0	26.48	43.09	61.13	232.26	376.90
1-Decanol		0	0	0	19.02	26.12	25.77
alpha.-Terpineol		0	0	0	0	18.67	31.53
2,4-Di-tert-butylphenol		40.93	85.16	38.18	67.32	21.72	20.49
n-Nonadecanol-1		0	0	0	0	18.67	40.69
2-Butenal	*Aldehyde*	28.46	0	0	0	0	0
Hexanal		54.28	35.19	30.22	32.71	38.09	20.40
Heptanal		19.98	0	0	0	0	0
Octanal		0	31.72	0	0	0	0
Benzeneacetaldehyde		0	0	0	0	0	34.60
2-Octenal, (E)		33.83	0	0	0	0	0
Nonanal		21.53	20.68	39.91	20.02	59.60	48.36
Decanal		29.85	20.10	33.40	24.82	30.38	40.58
Benzaldehyde, 2,4-dimethyl-		0	0	0	0	158.41	22.23
Benzaldehyde, 4-propyl-		0	0	19.55	28.18	0	0
Tetradecanal		20.11	0	0	0	0	0
Pentadecanal		206.19	232.60	121.37	52.28	41.96	66.45
Hexadecanal		0	0	53.55	30.59	34.86	37.77
9,17-Octadecadienal, (Z)		35.517	0	0	0	0	0
cis-9-Hexadecenal		20.073	0	0	0	0	0
Decane	*Alkane*	0	19.08	0	0	0	0
3-Ethyl-3-methylheptane		0	0	19.65	0	0	0
Dodecane, 4,6-dimethyl-		19.14	37.05	30.33	30.45	35.27	37.75
Nonane, 5-butyl-		0	0	0	0	26.98	0
Dodecane, 2,6,10-trimethyl-		0	0	21.16	0	0	0
Tetradecane		0	0	0	27.64	0	0
Eicosane		26.28	30.44	25.37	34.09	33.19	37.08
Heptadecane		19.76	19.79	19.82	19.98	19.63	19.87
Heneicosane		40.68	19.71	45.71	71.58	45.46	40.98
Dotriacontane		0	0	0	0	32.52	0
Octanoic acid	*Fatty acid*	0	34.79	28.83	20.16	0	0
Nonanoic acid		0	28.01	40.21	0	0	0
n-Decanoic acid		0	0	43.78	99.84	110.74	190.57
Dodecanoic acid		0	19.519	20.314	86.618	0	0
Tetradecanoic acid		27.81	35.99	157.78	33.52	19.77	32.44
n-Hexadecanoic acid		125.15	92.67	263.38	751.87	36.02	950.85
Oleic Acid		0	21.59	0	0	0	0
Methyl acetate	*Ester*	0	0	0	0	36.51	31.651
Ethyl Acetate		0	31.71	62.80	29.58	200.82	7842.38
Acetic acid, 2-phenylethyl ester		0	0	0.00	19.62	20.42	62.18
Diethyl Phthalate		54.25	32.87	20.36	0.00	34.85	0.00
Nonadecanoic acid, ethyl ester		0.00	0.00	19.65	0	0.00	0
Phthalic acid, hept-3-yl isobutyl ester		0	19.05	20.01	28.51	19.1	0
Hexadecanoic acid, methyl ester		21.75	19.57	0	0.00	19.93	35.43
Ethyl 9-hexadecenoate		0.00	0.00	0	0	39.93	20.50
Hexadecanoic acid, ethyl ester		20.56	20.79	26.64	25.65	20.86	27.69
Benzene, 1,2,3,5-tetramethyl-	*Hydrocarbon*	20.15	34.54	33.14	21.28	0.00	0.00
Benzene, 1-ethyl-3,5-dimethyl-		19.94	0.00	19.74	37.22	0	0
Benzene, 1,3-bis(1,1-dimethylethyl)-		41.68	42.03	19.74	20.59	0	0
Naphthalene		241.71	26.67	158.40	33.50	35.26	0
Naphthalene, 1-methyl-		171.11	224.86	186.40	385.30	0.00	0
Naphthalene, 2-methyl-		88.61	23.37	22.45	157.11	0	0
Naphthalene, 1,3-dimethyl-		19.47	34.03	19.90	35.62	0	0
Benzene, pentamethyl-		0.00	0.00	19.43	0.00	0	0
1,3,5,7-Cyclooctatetraene		0	33.39	46.52	19.46	24.97	215.16
Benzene, 1-methoxy-4-methyl-		0	0	0	0.00	32.23	0.00
2-Heptanone	*Ketone*	20.06	18.9	0	0	0	0
2-Octanone		32.97	38.14	20.78	20.37	0	0
2-Nonanone		31.64	34.86	50.76	41.31	0	0
Isophorone		19.44	19.54	17.02	42.89	0	0
2-Decanone		0	0	25.04	18.67	0	0
5,9-Undecadien-2-one, 6,10-dimethyl-, (*E*)-		0	0	0	19.64	42.24	19.574
2H-Pyran, 3,6-dihydro-4-methyl-2-(2-methyl-1-propenyl)	*Pyran*	0	0	0	0.00	19.85	32.16
2,3’-Bifuran, octahydro	*Furan*	19.30	0	0	0	0	0

At 0 h, aldehydes (e.g., pentadecanal), fatty acids (e.g., hexadecanoic acid), and hydrocarbons (e.g., naphthalene and 1-methyl naphthalene) were predominant in coffee pulp. Their concentrations progressively declined during fermentation as a result of microbial transformations, including reduction, oxidation, β-oxidation, and oxidative degradation catalyzed by enzymes such as peroxidases, monooxygenases, and laccases (da Vale et al., 2024; van Mullem et al., 2022). In particular, LAB, which dominate coffee fermentations, actively contribute to the removal of hydrocarbons and other aromatic compounds through metabolic activity and surface adsorption mechanisms (Shao et al., 2022).

After 24 h of fermentation, the bioconversion of precursors into alcohols and esters became evident and dominated the shift in the volatile profile. Linalool and phenylethyl alcohol emerged as the main alcohols, reaching 475.45 and 376.90 μmol L^−1^, respectively, by the end of fermentation. Linalool formation is consistent with yeast-driven flux through the mevalonate (isoprenoid) pathway that supplies monoterpene biosynthesis, whereas phenylethyl alcohol arises via the yeast Ehrlich pathway from phenylalanine (de Pereira et al., 2019; Dzialo et al., 2017; Kallscheuer et al., 2019). In contrast, studies from other origins scarcely report linalool and phenylethyl alcohol as predominant fermentation-phase volatiles: in northern Peru (washed; short vs. long fermentations), key differentiators were benzaldehyde, methional, hexanal, 2-heptanone and esters, such as isoamyl acetate and 2-phenylethyl acetate, rather than monoterpenes (Perez et al., 2023); in Brazil, yeast inoculation primarily increased higher alcohols (e.g., isoamyl alcohol) and esters, while bacterial treatments favored organic acids (Martinez et al., 2019); and in Australian wet fermentations, suppressing yeasts mainly reduced ethanol, isoamyl alcohol and ethyl acetate rather than monoterpenes (Elhalis et al., 2021; Elhalis, Cox, Frank, & Zhao, 2020). Taken together, the unusually high linalool and phenylethyl alcohol observed here indicate particularly active yeast metabolism, which may contribute to the development of floral and fruity notes in the final beverage (Elhalis, Cox, Frank, & Zhao, 2020; Martinez et al., 2019). Other alcohols formed included 3-methyl-1-butanol, 2-heptanol, 1-hexanol, 1-octen-3-ol, and 2,4-di-tert-butylphenol, consistent with pronounced amino-acid and lipid catabolism (de Pereira et al., 2019).

Esters were the second most abundant chemical class, led by ethyl acetate, which peaked at 7842.38 μmol L^−1^ after 72 h of fermentation. Short-chain esters (e.g., methyl acetate and phenyl acetate) began accumulating from 36 h, and long-chain fatty-acid esters (e.g., methyl hexadecanoate, ethyl 9-hexadecenoate, and ethyl hexadecanoate) appeared from mid- to late fermentation. This timing reflects sequential precursor availability and phase-specific yeast regulation: early acetyl-CoA and Ehrlich-derived higher alcohols favor short-chain acetate esters, whereas later ethanol and fatty acyl-CoA favor medium/long-chain fatty-acid ethyl esters (Dzialo et al., 2017). These esters confer fruity and sweet notes to fermented coffee. Comparable ester accumulations have been documented in other origins—ethyl acetate frequently predominates, with short-chain esters (e.g., methyl/isoamyl/2-phenylethyl acetate) increasing over 36–72 h and long-chain fatty-acid ethyl esters (e.g., ethyl palmitate, and ethyl 9-hexadecenoate) emerging during extended fermentations (De Bruyn et al., 2017; Elhalis et al., 2021; Martinez et al., 2019; Perez et al., 2023).

### 3.2 Metagenetic profiling of coffee fermentation

**Fig. 2:**
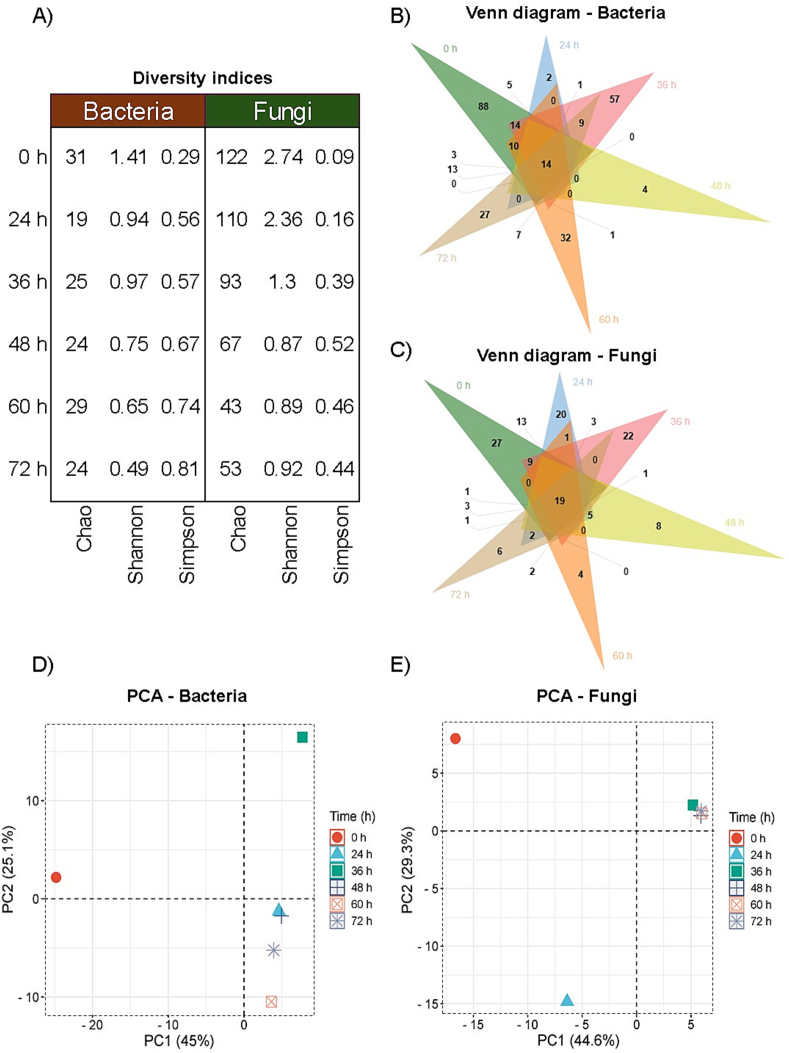
Diversity indices (Chao, Shannon, and Simpson) (A), Venn diagrams (B and C), and principal component analysis (D and E) of bacterial and fungal communities identified during coffee bean fermentation carried out in Chimanimani National Park, Mozambique.

A total of 1,789,909 high-quality sequences passed the QIIME quality filters and were taxonomically assigned to 332 bacterial and 202 fungal genera, with rarefaction and coverage analyses confirming sufficient sequencing depth (Table S1, Fig. S3). A total of 88 bacterial and 27 fungal OTUs were exclusively detected at the initial point of fermentation (Fig. 2B-C), reflecting a unique microbial signature shaped by the local environment. As fermentation progressed, this number decreased significantly, with only 33 predominant OTUs (19 fungal and 14 bacterial genera) remaining at the end of the process. This dynamic was confirmed by the behavior of alpha diversity indices, including the Chao1 and Shannon indices (richness and evenness), and the Simpson's index (dominance), all of which indicated a progressive reduction in community diversity (Fig. 2A). In line with these results, Principal Component Analysis (PCA) revealed a clear temporal structuring of the microbial communities, with distinct clustering of samples according to fermentation time (Fig. 2D-E). These findings suggest that microbial succession during coffee fermentation is a highly selective process, driven by environmental pressures and microbial interactions (Pothakos et al., 2020). This succession represents the central axis of ecological modulation in coffee fermentation and can be utilized to develop targeted strategies for microbial management and process control.

At the onset of fermentation, the bacterial community was predominantly composed of *Vibrio* (36.04 %), *Leuconostoc* (34.42 %), and *Pantoea* (21.87 %), while the fungal community exhibited a more even distribution among several genera, including *Vishniacozyma* (15.96 %), *Papiliotrema* (13.62 %), *Hannaella* (11.70 %), *Galactomyces* (11.28 %), *Cladosporium* (10.11 %), *Pichia* (9.11 %), *Cercospora* (5.15 %), *Hanseniaspora* (4.15 %), and *Candida* (2.61 %) (Fig. 1). This initial microbial richness likely reflects multiple sources of contamination, including the environment, coffee cherries, and post-harvest handling conditions (da Vale et al., 2021; Pregolini et al., 2021; Zhang et al., 2022).

The fermentation process was rapidly dominated by well-adapted fermentative species, with *Leuconostoc* peaking at 89.99 % by 72 h. Among yeasts, a clear trade-off was observed between *Hanseniaspora* and *Galactomyces*; while *Hanseniaspora* peaked at 67.63 % at 48 h, *Galactomyces* gradually increased and became dominant by the final stage of fermentation (44.82 % at 72 h). Other microbial genera, such as *Weissella* and *Pichia*, also emerged at specific times with relative abundances exceeding 5 %, playing secondary roles in the microbial ecosystem.

To the best of our knowledge, this is the first time that the co-occurrence of *Hanseniaspora* and *Galactomyces* has been observed in coffee fermentation or any other known fermentative system. In coffee fermentations, *Pichia* is frequently reported as the dominant yeast, particularly in processes conducted in Colombia, Ecuador, and Australia (De Bruyn et al., 2017; de Junqueira et al., 2019; Elhalis, Cox, Frank, & Zhao, 2020; Zhang et al., 2022). This contrast underscores the potential influence of the unexplored Chimanimani *terroir* encompassing native microbial reservoirs, local environmental conditions, and post-harvest practices on shaping a distinct yeast community structure. Furthermore, the possibility of a synergistic interaction between *Hanseniaspora* and *Galactomyces* should be explored, as similar relationships involving metabolite cross-feeding and mutual tolerance to environmental stressors have been described in other ecological systems (Ponomarova & Patil, 2015). *Hanseniaspora* is widely recognized for its ability to produce high levels of esters, contributing to fruity and floral notes (Filippousi et al., 2024; Valera et al., 2024), while *Galactomyces* has been associated with buttery, creamy, and honey-like flavors in fermented foods such as dairy products and cocoa (Szudera-Kończal et al., 2021).

**Fig. 3:**
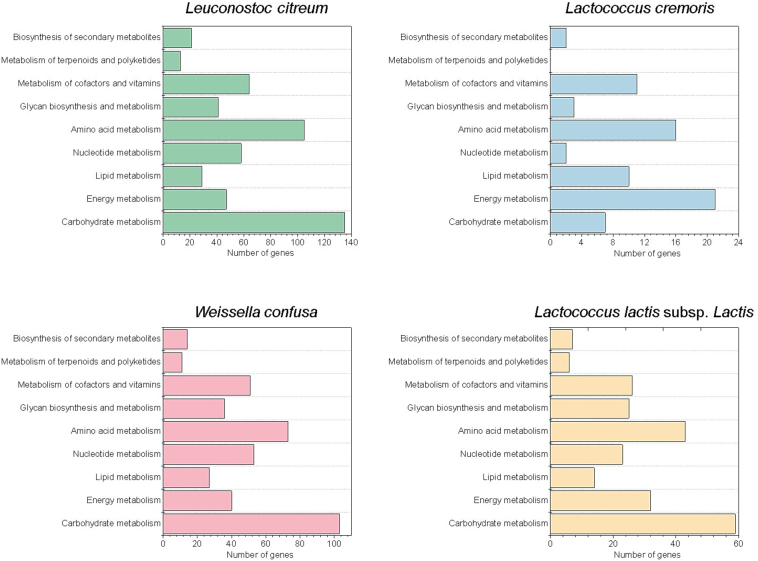
Functional classification of genes annotated in genomes assembled from metagenomes (MAGs) of four bacterial species identified in coffee fermentation. The graphs show the number of genes associated with the main metabolic pathways, highlighting the differences in functional potential between *Leuconostoc citreum*, *Lactococcus cremoris*, *Weissella confusa*, and *Lactococcus lactis* subsp. *lactis*.

Although commonly found in coffee fermentations, *Leuconostoc* is typically outcompeted over time by *Lactobacillus,* which shows greater tolerance to acidic stress (da Vale et al., 2021; da Vale et al., 2024; De Bruyn et al., 2017; de Junqueira et al., 2019; Peñuela-Martínez et al., 2023; Pothakos et al., 2020). However, in this study, *Leuconostoc* remained dominant until the final stage of fermentation. To further investigate this outcome, we performed metagenomic assembly using shotgun sequencing data obtained at the end of fermentation. This approach enabled the reconstruction of four high-quality metagenome-assembled genomes (MAGs) with ≥80 % completeness and ≤10 % contamination, corresponding to *Leuconostoc citreum*, *Lactococcus cremoris*, *Weissella confusa*, and *Lactococcus lactis* subsp. *Lactis* (Fig. 3).

Functional annotation of these genomes revealed that *Leuconostoc citreum* possessed a remarkably enriched set of genes related to key metabolic pathways, including carbohydrate metabolism (135 genes), amino acid metabolism (105), nucleotide metabolism (58), energy metabolism (47), and cofactor and vitamin biosynthesis (64). Compared to other recovered MAGs, *Leuconostoc* exhibited the highest number and percentage of genes in almost all core metabolic categories, suggesting a superior metabolic versatility and adaptability. These functional capabilities may have enabled *Leuconostoc* to rapidly utilize available substrates, withstand environmental stressors, and outcompete other species under the specific conditions of the fermentation system in Chimanimani. This ecological advantage likely explains its sustained dominance, contrasting with trends observed in other coffee-producing regions.

### 3.3 Shotgun metagenomics

#### 3.3.1 Metabolic pathways and functional genes driving coffee fermentation chemistry

**Fig. 4:**
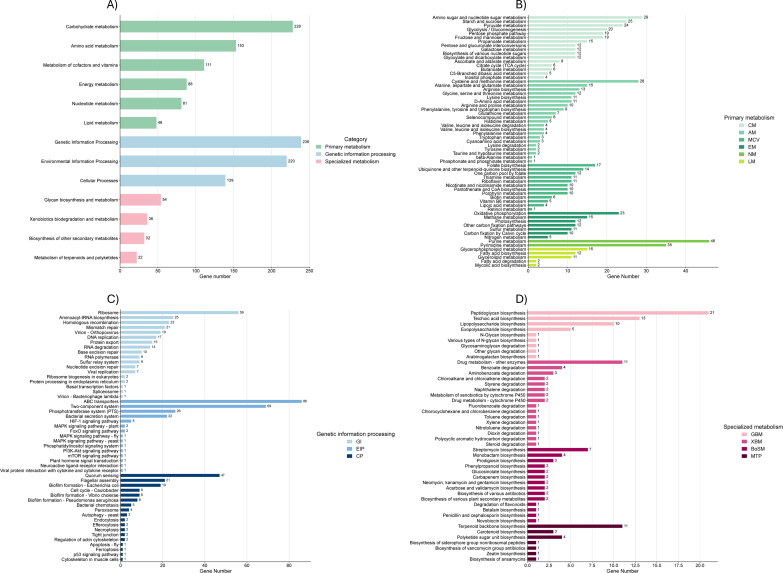
Functional classification of annotated genes based on KEGG metabolic pathways during coffee bean fermentation in Chimanimani National Park, Mozambique. (A) Overall distribution of the number of genes by functional category, (B) genes associated with primary metabolism, (C) genes related to genetic information processing, and (D) genes involved in specialized metabolism. Pathways reconstructed using the KEGG database (Kanehisa et al., 2025).

In total, 234 metabolic pathways comprising 1791 functional genes were identified in the coffee fermentation microbiome, falling into three broad categories: primary metabolism (703 genes), genetic information processing (598 genes), and specialized metabolism (122 genes) (Fig. 4A). Within primary metabolism, carbohydrate metabolism was the dominant functional class (228 genes), underscoring the central role of sugar utilization in driving the fermentative process. The most represented pathways were amino and nucleotide sugar metabolism (29 genes), starch and sucrose metabolism (25 genes), and glycolysis/gluconeogenesis (20 genes) (Fig. 4B).

The amino and nucleotide sugar metabolism gene set indicates a strong capacity for sugar interconversion and activation in the microbial community. Enzymes like UDP-glucose pyrophosphorylase and various UDP-hexose epimerases can convert basic sugar phosphates into activated forms (e.g. UDP–glucose, UDP–galactose, UDP–GlcNAc). These activated sugars serve as precursors for building structural carbohydrates in cells. In yeasts and LAB, such nucleotide sugars fuel cell-wall polysaccharide biogenesis (e.g., chitin in yeast cell walls uses UDP-GlcNAc) and the synthesis of exopolysaccharides (EPS) (De Bruyn et al., 2017; Pothakos et al., 2020). In summary, the high presence of this gene set suggests these coffee pulp microorganisms can efficiently interconvert sugars and activate them for biosynthetic purposes, reinforcing their ability to thrive in the pulp by constructing necessary cell components and protective EPS matrices.

Consistent with the sugar-rich pulp matrix (Fig. 1), starch/sucrose metabolism (25 genes) included invertase/β-fructofuranosidase (EC 3.2.1.26), sucrase-isomaltase, and sucrose phosphorylase (EC 2.4.1.7) activities typically associated with early yeast colonizers that hydrolyze sucrose into fermentable monosaccharides. These simple sugars are rapidly utilized by yeasts themselves and simultaneously provide substrates for co-occurring LAB, which convert hexoses into organic acids that modulate succession and flavor development (De Bruyn et al., 2017; Gänzle, 2015). The detection of invertase, sucrase-isomaltase, and sucrose phosphorylase genes is therefore consistent with a typical yeast–LAB synergy driving early stages of coffee pulp fermentation. Thus, in our dataset, the high prevalence of starch/sucrose metabolism genes highlights sucrose from pulp mucilage and starch reserves as the main carbon source, explaining the dominance of yeasts and LAB in coffee fermentations.

Interestingly, the preferential consumption of fructose observed in the chemical analyses (Fig. 1) was further supported by metagenomic data, which revealed a high abundance of genes related to fructose transport and metabolism, such as fructose-specific permeases and fructokinase (Fig. 4B). These genes are typically associated with *Hanseniaspora* (Dzialo et al., 2017), reinforcing its functional role in driving early sugar assimilation during fermentation. In addition*, Leuconostoc* may have complemented this metabolic activity, as it possesses fructose-specific phosphotransferase systems (PTS) and can utilize fructose both as a fermentable sugar and as an alternative electron acceptor for mannitol production, contributing to redox balance and potentially influencing metabolite profiles (Gänzle, 2015).

Amino acid metabolism was the second most represented primary metabolic pathway, with 153 genes identified (Fig. 4A). Genes involved in the metabolism of cysteine, methionine, alanine, aspartate, glutamate, arginine, glycine, threonine, and phenylalanine were detected (Fig. 4B). These pathways play essential roles in microbial physiology by enabling protein biosynthesis, maintaining redox balance, enhancing stress tolerance, and generating metabolic precursors required for growth and adaptation during fermentation (Liu et al., 2013). For coffee, many of the secondary metabolites derived from amino acid catabolism, such as higher alcohols, aldehydes, and esters, are key contributors to the formation of floral, fruity, and sweet notes that directly impact the sensory attributes of the final beverage (Dzialo et al., 2017).

Lipid metabolism genes were detected at low abundance (48 genes, Fig. 4A), consistent with the low lipid content of coffee pulp and comprising functions related to fatty acid β-oxidation and glycerophospholipid biosynthesis. These functions likely support microbial membrane maintenance, with cells adjusting lipid composition to withstand variations in pH, temperature, and metabolites during fermentation. The presence of fatty acid degradation genes suggests microbes could scavenge any available lipids (from the pulp or dead cells) for energy or membrane remodeling. Likewise, genes for phospholipid biosynthesis imply active cell membrane synthesis to support rapid cell growth and possibly biofilm formation. Although lipid metabolism played a minor role in this system, its occurrence aligns with recent findings by Morales-Palomo et al. (2024) that even in carbohydrate-rich fermentations, foundational lipid metabolic processes persist to maintain cellular integrity under stress.

Pathways related to genetic information processing (aminoacyl-tRNA biosynthesis, ribosomal assembly, DNA repair and replication mechanisms, and protein secretion), environmental interaction (phosphotransferase system [PTS] and bacterial secretion systems), and cellular processes (biofilm formation, flagellar assembly, and chemotaxis) were all highly represented (Fig. 4A–C). Together, these three functional categories highlight a metabolically active and well-organized microbiome capable of adapting to the fermentative environment, efficiently assimilating nutrients, and employing colonization strategies that promote process stability and performance.

In addition, at the level of specialized metabolism (Fig. 4A), pathways associated with the biosynthesis of secondary metabolites (32 genes), glycans and biopolymers (54 genes), and xenobiotic metabolism (36 genes) were observed. Although less abundant in terms of the number of genes, these functions support the stability and resilience of the fermentative microbiota through the production of bioactive compounds and mechanisms to degrade complex molecules. These specialized functions contribute to microbial adaptation under stressful conditions while also enhancing the functional and sensory differentiation of fermented coffee. This reinforces the importance of exploring microbial biodiversity and metabolic potential in origin-specific fermentation processes.

#### 3.3.2 Microbial pathways linking substrate utilization to volatile and flavor compound production in coffee fermentation

**Fig. 5:**
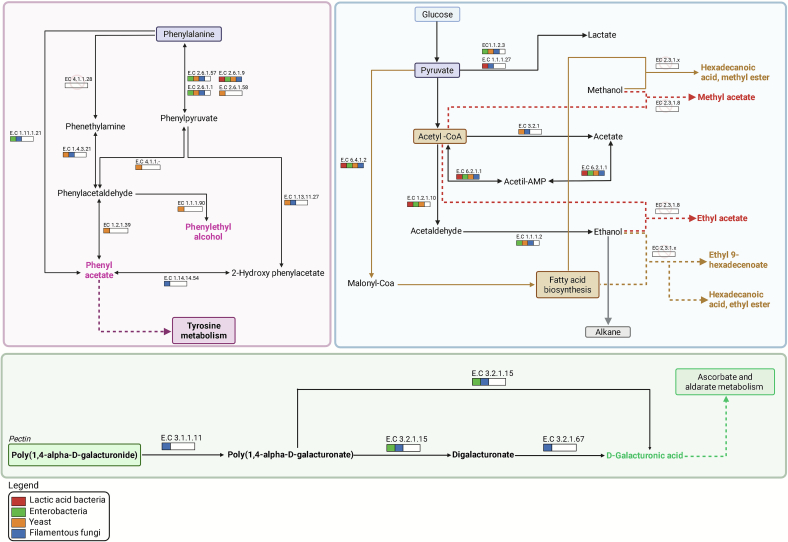
Reconstruction of microbial metabolic pathways associated with the formation of volatile compounds during coffee bean fermentation, based on functional annotation of genes by the KEGG and eggNOG databases. The main pathways involved in phenylalanine metabolism, sugar and pyruvate derivative metabolism, and pectin degradation are shown. Enzymes are identified by their respective EC codes and colored according to the predominant microbial groups: lactic acid bacteria (red), enterobacteria (green), yeasts (yellow), and filamentous fungi (blue). Pathways reconstructed using the KEGG database (Kanehisa et al., 2025). (For interpretation of the references to colour in this figure legend, the reader is referred to the web version of this article.)

To guide the focused metabolic reconstruction, three pathways were prioritized based on their direct relevance to the dominant biochemical transformations observed during fermentation (Fig. 5). First, phenylalanine catabolism was examined because phenylalanine serves as the precursor of phenylethyl alcohol, the most abundant microbial-derived volatile detected in this study. Second, central carbon metabolism associated with ester biosynthesis was included, as esters represented the most sensorially impactful class of fermentation-related VOCs and are tightly linked to yeast carbohydrate metabolism. Third, pectin degradation pathways were investigated due to their importance in mucilage breakdown, which influences microbial succession, substrate accessibility, and the overall progression of coffee fermentation. These pathways illustrate the coordinated activity of LAB (marked in red), enterobacteria (green), yeasts (orange), and filamentous fungi (blue) in transforming coffee pulp substrates into volatile and bioactive compounds, thereby supporting both flavor development and microbial adaptation throughout the fermentation process (Fig. 5).

Functional analysis of the enzymes associated with phenylalanine metabolism revealed the activation of the Ehrlich pathway, a classic route described in fermentative yeasts, responsible for converting aromatic amino acids into higher alcohols and volatile acids (Hazelwood et al., 2008). Phenylalanine is initially converted into phenylpyruvate by aminotransferases, whose genes have been annotated as EC 2.6.1.57, EC 2.6.1.9, EC 2.6.1.1, and EC 2.6.1.58 This initial step showed a wide distribution among different microbial groups, indicating that phenylalanine transamination can occur cooperatively or redundantly in the fermentation community. On the other hand, the subsequent steps of the pathway showed a predominant association with yeasts; phenylpyruvate is converted into phenylacetaldehyde by decarboxylases of the EC 4.1.1.- class, followed by two possible routes: its reduction to phenylethyl alcohol by phenylacetaldehyde reductase (EC 1.1.1.90) or its oxidation to phenyl acetate by phenylacetaldehyde dehydrogenase (EC 1.2.1.39). These results confirm that yeasts are the main contributors to the formation of phenylethyl alcohol, a key floral-active compound, underscoring their central role in shaping the aromatic profile of fermented coffee.

From pyruvate, the conversion into acetyl-CoA represents a central point for the synthesis of esters during fermentation. Among the products formed are simple esters, such as ethyl acetate and methyl acetate, and fatty acid esters, such as ethyl 9-hexadecenoate, hexadecanoic acid ethyl ester, and hexadecanoic acid methyl ester. All these compounds, identified in the fermentation liquid fraction (Table 1), are formed from reactions between acetyl-CoA or fatty acids and alcohols, such as ethanol or methanol, catalyzed by different classes of acyltransferases. The formation of simple esters is attributed to the activity of alcohol *O*-acetyltransferases (EC 2.3.1.8), encoded by genes such as ATF1 and ATF2, while the synthesis of fatty acid esters is associated with the action of acyl-CoA: alcohol O-acyltransferases (EC 2.3.1.x), represented by the EHT1 and EEB1 genes in fermentative yeasts (Dzialo et al., 2017). Although these enzymes were not directly annotated in the metagenomic data by the KEGG and eggNOG databases, the consistent presence of the resulting compounds suggests the action of other functionally similar enzymes, not yet characterized or recovered by the annotation tools used.

Multiple studies have reported pectinolytic activity by microorganisms during coffee and cocoa fermentations, including yeasts (e.g., *Candida boidinii*, *Pichia kudriavzevii*) and bacteria (e.g., *Erwinia* spp., *Enterobacter* spp.) (Ardhana, 2003; Avallone et al., 2002; Masoud & Jespersen, 2006). However, our findings, consistent with key literature, indicate that microbial mucilage degradation in coffee may be considerably limited (Avallone et al., 2001; Avallone et al., 2002). Avallone et al. (2002) observed that wet-fermented coffee mucilage underwent only slight pectin de-esterification without meaningful depolymerization, likely due to the mucilage's high degree of methylation. In agreement, the only pectinolytic isolates they recovered were *Erwinia herbicola* and *Klebsiella pneumoniae* (Enterobacteriaceae), both weak pectin degraders. Cocoa fermentation studies further support this pattern: early-stage pectin breakdown is predominantly driven by Enterobacteriaceae (e.g., *Enterobacter*), whereas yeasts and lactic acid bacteria mainly ferment sugars and contribute little to pectin hydrolysis (Ardhana, 2003).

In our study, pathway-level reconstruction revealed the conversion of polygalacturonides into digalacturonate and D-galacturonic acid, with links to ascorbate metabolism. The enzymes implicated in this process, such as pectin methylesterase (EC 3.1.1.11), were primarily associated with filamentous fungi and enterobactéria (Fig. 5). However, these groups underwent a drastic decline within the first 24 h of fermentation (Fig. 1), making their effective involvement in the initial stages of mucilage degradation unlikely.

Aligned with these observations, our 72 h metagenomic profile revealed no detectable genes encoding polygalacturonases, pectin lyases, or pectin esterases among the dominant microbiota. This coincided with a marked decline in filamentous fungi, enterobacteria, and known pectinolytic yeasts, taxa typically associated with pectinase activity. Taken together, the absence of microbial pectinolytic signatures at 72 h, combined with our multi-omics evidence, led us to hypothesize that mucilage removal in this fermentation was driven largely by endogenous coffee enzymes rather than by microbial action.

Therefore, our interpretation that endogenous bean enzymes primarily facilitated mucilage degradation is presented as a plausible scenario supported by current evidence rather than a definitive conclusion. Further studies incorporating early time-point sampling and direct pectinase activity surveys will be essential to disentangle microbial and plant-derived contributions, as also recommended in previous work.

### 3.4 Chemical composition and sensory attributes of fermented coffee beans

**Fig. 6:**
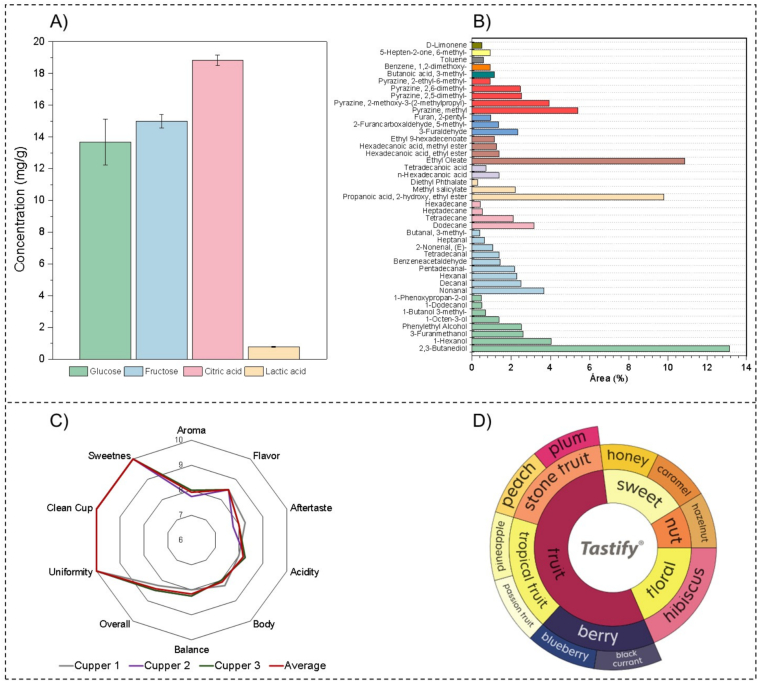
Chemical composition of green coffee beans and sensory attributes of the beverage. (A) Average concentrations of glucose, fructose, citric acid, and lactic acid. (B) Relative abundance of volatile compounds (% of total chromatographic area). (C) Sensory scores assigned by certified Q-Graders. (D) Descriptive flavor profile based on the sensory wheel. (For interpretation of the references to colour in this figure legend, the reader is referred to the web version of this article.)

As illustrated in Fig. 6, chemical and sensory profiling of the fully washed coffee beans highlighted the clear imprint of microbial metabolism on the final product. Phenylethyl alcohol, the major volatile compound produced during fermentation (Table 1), was also observed in expressive amounts in the green beans (2.5 %, Fig. 6B). Its accumulation during fermentation and association with a characteristic rose-like aroma provide direct evidence that microbial metabolism contributed to the volatile reservoir later expressed in the beverage (Fig. 6 C and D). In addition to phenylethyl alcohol, other fermentation-derived metabolites were detected at higher relative abundances, including 2,3-butanediol (13.03 %), resulting from sugar fermentation by yeasts and LAB, and ethyl lactate (9.08 %), formed through esterification of lactic acid and ethanol produced by LAB and yeasts, respectively. Interestingly, the concomitant presence of lactic acid and ethyl lactate suggests that lactic acid absorbed during fermentation was further transformed into esters during drying, most likely via residual esterase or alcohol acyltransferase activity still present in the beans (Bornscheuer, 2002). The exclusive presence of ethyl lactate in the green beans and its absence in the liquid fraction reinforces the hypothesis that its formation occurred inside the bean during drying, contributing to the final aromatic profile of the coffee.

Together, these compounds represent a microbial biochemical signature and contribute to floral, fruity, and sweet attributes in the final cup (Fig. 6D). In parallel, several volatiles typically linked to plant metabolism were also detected, such as benzeneacetaldehyde (1.44 %), 1-hexanol (4.0 3%), hexanal (2.28 %), and nonanal (3.65 %), associated with floral, herbaceous, and fruity notes. These compounds reflect the intrinsic metabolic fingerprint of the coffee fruit, shaped by variety, soil, and climatic conditions, and thus contribute to the *terroir* identity (da Tapaça et al., 2024; da Vale et al., 2024).

Primary metabolites quantified in the beans further supported their contribution to flavor development (Fig. 6A). Glucose (13.68 ± 1.44 mg/g) and fructose (17 ± 0.42 mg/g) were present in significant amounts, serving as key precursors for Maillard and caramelization reactions during roasting. Among the organic acids, citric acid (18.82 ± 3.03 mg/g) was the most abundant and represents an endogenous fruit metabolite, while lactic acid (0.78 ± 0.03 mg/g), primarily of microbial origin, confirmed LAB activity during fermentation and positively influences sensory perception by enhancing acidity, body, and smoothness in the cup (de Pereira et al., 2020).

The roasted beverage was classified as specialty coffee and scored 87.25 ± 0.25 points under the SCA protocol, with consistently high ratings for aroma, flavor, sweetness, aftertaste, and balance (Fig. 6C). The sensory evaluation revealed a broad spectrum of descriptors. This diversity reflects the combined action of microbial fermentation, endogenous bean chemistry, and thermal transformations during roasting, with pyrazines, furans, esters, and ketones contributing to complexity. The pronounced floral and red-fruit character (Fig. 6D) resembles the aromatic identity of high-altitude African coffees, known for their vibrant acidity (Demianova et al., 2022). Given that coffee cultivation in Mozambique is still emerging, these results highlight a promising potential to produce high-quality specialty coffees with distinctive sensory identity and added market value.

## 4 Conclusions

This study provides the first multi-omics characterization of coffee fermentation in an African region, revealing the interplay between microbial consortia, bean chemistry, and fermentation-driven metabolites in shaping cup quality. Shotgun metagenomics uncovered 1791 functional genes distributed across 234 metabolic pathways, confirming that sugar catabolism, amino acid transformations, and ester biosynthesis are central to flavor development. A core microbial axis formed by *Leuconostoc*, *Hanseniaspora*, and *Galactomyces*, not previously reported in coffee fermentations, was identified as a core consortium driving these biochemical routes.

The accumulation of phenylethyl alcohol (rose-like aroma) and linalool (floral aroma) inside the beans, together with the presence of ethyl acetate and other microbial esters, highlights their role as potential biochemical markers of African fermented coffees. These metabolites, combined with terroir-derived plant volatiles, contributed to a specialty-grade beverage (87.25 ± 0.25 points) characterized by distinctive fruity and floral notes, consistent with the high-altitude terroirs of Eastern Africa.

Overall, our findings demonstrate that a distinctive microbial–metabolite–terroir axis decisively shapes the sensory identity of Mozambican Arabica coffee. The elevated levels of phenylethyl alcohol and linalool highlight these compounds as promising microbial markers of fermentation-driven quality, forming a biochemical signature not previously reported for any other coffee-producing region. These newly identified microbial–chemical signatures are relevant not only for advancing our understanding of African coffee fermentation but also for informing the development of region-tailored microbial starter cultures and for establishing scientifically grounded criteria for the geographical differentiation of African specialty coffees. Although shotgun metagenomics revealed the functional potential of the coffee fermentation microbiome, future studies will benefit from integrating temporal metatranscriptomic approaches to capture real-time metabolic activity, particularly during the early and mid-fermentation stages, where *Hanseniaspora*-driven ester production and other transient biochemical processes peak. Future studies covering multiple harvest seasons, micro-regions, and independent fermentation batches across Africa will be essential to assess the robustness of these markers, confirm their reproducibility under diverse environmental conditions, and solidify the biochemical basis for recognizing and valorizing African specialty coffees in the global market.

## CRediT authorship contribution statement

**Gilberto Vinícius de Melo Pereira:** Writing – review & editing, Writing – original draft, Supervision, Resources, Methodology, Investigation, Conceptualization. **Alexander da Silva Vale:** Writing – review & editing, Validation, Methodology, Investigation, Formal analysis, Data curation. **Ana Isabel Ribeiro-Barros:** Writing – original draft, Resources, Funding acquisition, Conceptualization. **Luiz Roberto Saldanha Rodrigues:** Visualization, Validation, Formal analysis. **Gisela Manuela de França Bettencourt Mirção:** Writing – review & editing, Investigation. **Bernadete Camilo:** Writing – original draft, Investigation. **Inocência da Piedade Ernesto Tapaça:** Writing – review & editing, Investigation. **Vitoria de Mello Sampaio:** Writing – review & editing, Formal analysis, Data curation. **Satinder kaur Brar:** Writing – review & editing, Supervision. **Carlos Ricardo Soccol:** Writing – review & editing, Supervision, Resources, Project administration, Funding acquisition.

## Funding sources

This research work was financially supported by the 10.13039/501100003593National Council for Scientific and Technological Development, CNPq, grant nos. 440343/2022-4. AIR-B has received funds from FCT - Fundação para a Ciência e a Tecnologia, I.P. (UIDB/00239/2025 of Forest Research Center, DOI doi:10.54499/UIDB/00239/2020; LA/P/0092/2020 of Associate Laboratory TERRA, DOI doi:10.54499/LA/P/0092/2020).

## Declaration of competing interest

The authors declare that they have no known competing financial interests or personal relationships that could have appeared to influence the work reported in this paper.

## Data Availability

The datasets generated and/or analyzed during the current study are available in the NCBI repository under BioProject ID PRJNA1302651

## Appendix A Supplementary data

Complementary data on chemical, microbial, and fermentation analyses (Figures S1-S4; Tabke S1). Image 1
