# Supplementary material for: Integrated microbial–metabolomic analysis reveals how fermentation contributes to the unique flavor of African Arabica coffee
Source: Food Chem (Oxf). 2025 Dec 18;12:100344. doi: 10.1016/j.fochms.2025.100344 (PMC12859811; doi:10.1016/j.fochms.2025.100344)
Supplement: Supplementary file 1 — Complementary data on chemical, microbial, and fermentation analyses (Figures S1-S4; Tabke S1). [file mmc1.docx]

Supplementary Materials

**Integrated microbial–metabolomic analysis reveals how fermentation contributes to the unique flavor of African Arabica coffee**

Gilberto Vinícius de Melo Pereira^1†^, Alexander da Silva Vale^1†^, Ana Isabel Ribeiro-Barros^2*^, Luiz Roberto Saldanha Rodrigues^3^, Gisela Manuela de França Bettencourt Mirção^4^, Bernadete Camilo^4^, Inocência da Piedade Ernesto Tapaça^2^, Vitoria de Mello Sampaio^1^ Satinder kaur Brar^5^, Carlos Ricardo Soccol^1*^

^1^Federal University of Paraná (UFPR), Department of Bioprocess Engineering and Biotechnology, Curitiba, PR, Brazil.

^2^Forest Research Center, Associate Laboratory TERRA, School of Agriculture, University of Lisbon, Tapada da Ajuda, 1349-017 Lisbon, Portugal.

^3^Federal University of Technology Paraná (UTFPR), Department of Chemistry and Biology, Curitiba, PR 80230-901, Brazil.

^4^Higher Polytechnic Institute of Manica (ISPM), Division of Agriculture, Biotechnology Program, Manica, Mozambique.

^5^Department of Civil Engineering, Lassonde School of Engineering, York University, North York, Toronto, ON M3J 1P3, Canada


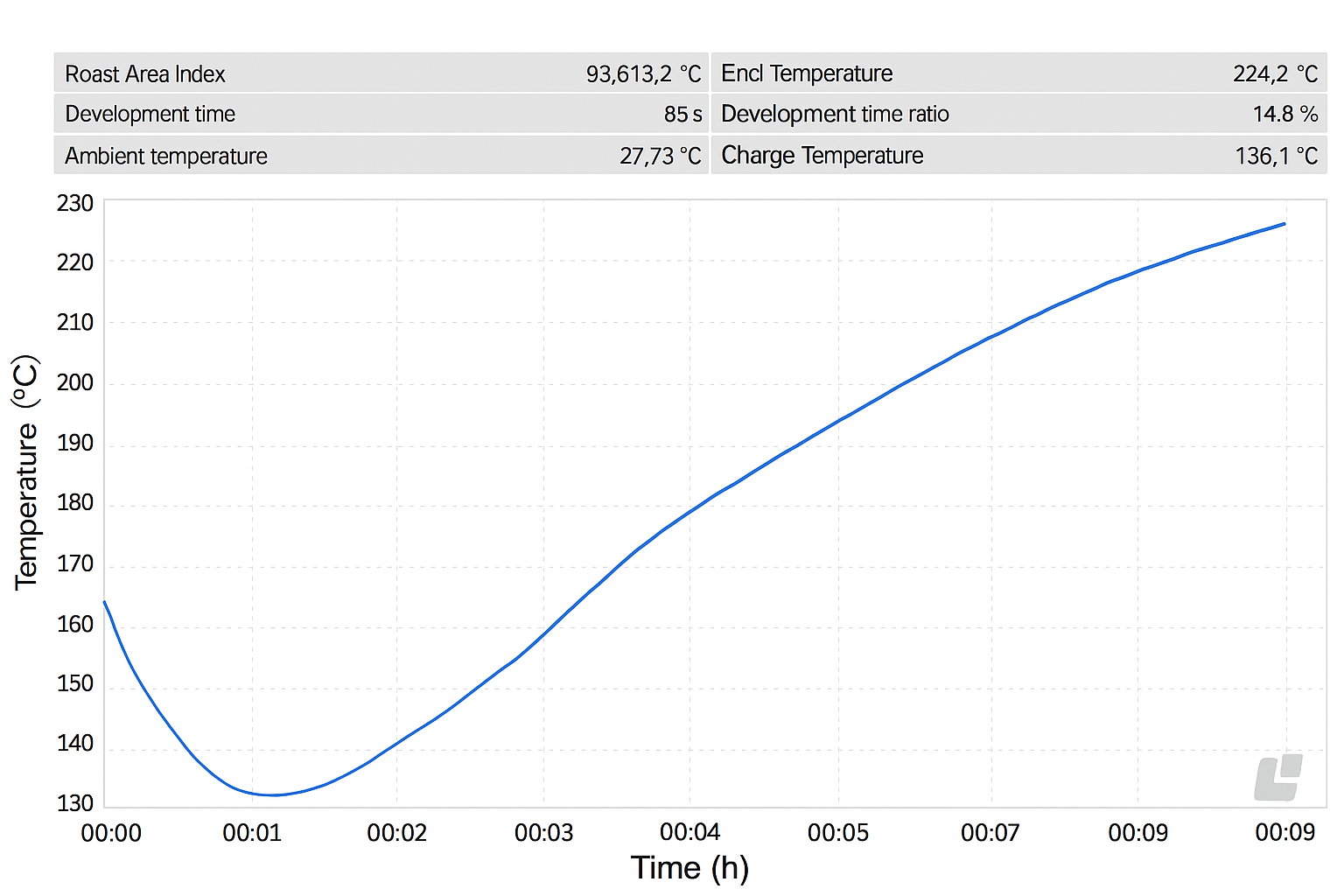


**Figure S1:** Roasting curve developed for coffee beans produced in Chimanimani National Park, Mozambique.

**
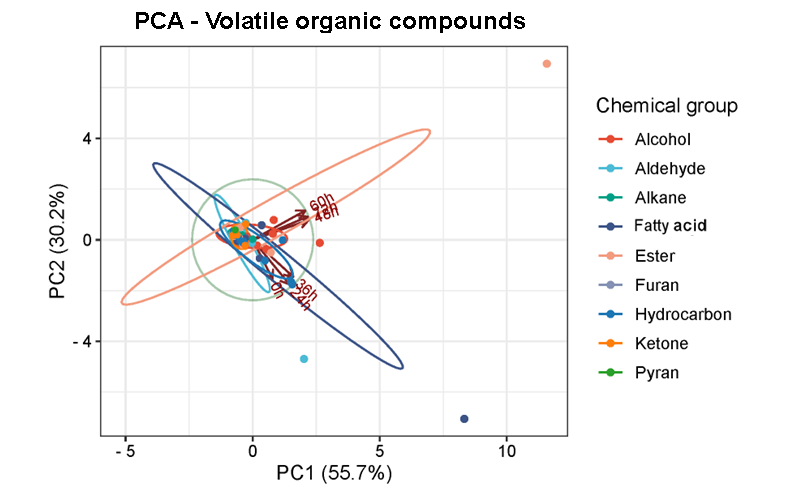
**

**Figure S2:** Principal component analysis (PCA) of volatile compounds identified in the liquid fraction during coffee fermentation.


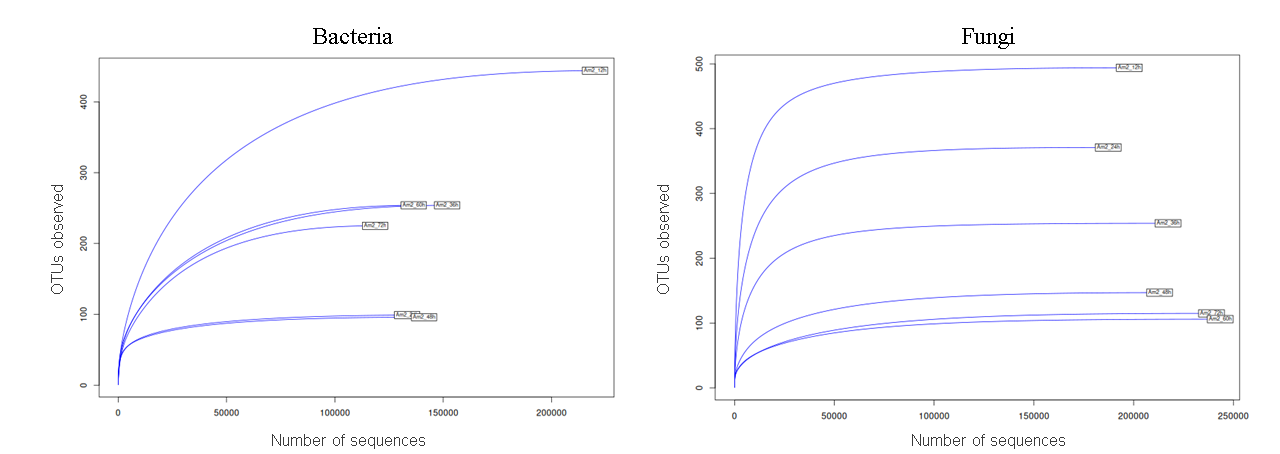


**Fig. S3:** Alpha rarefaction curves of OTUs (operational taxonomic units) observed in the liquid fraction of fermentation.


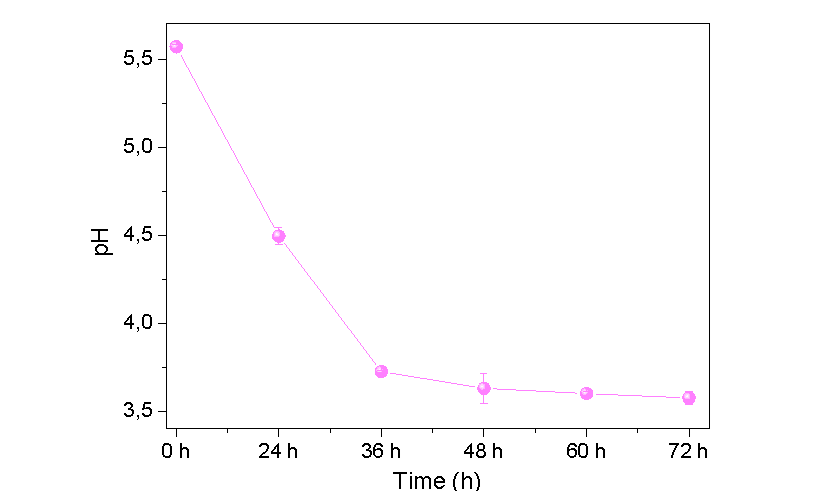


**Fig S4:** pH variation during the fermentation process over 72 hours.

**Supplementary table 1:** Relative abundance (%) of bacteria and fungi identified during fermentation

| **Genus** | **0 h** | **24 h** | **36 h** | **48 h** | **60 h** | **72 h** |
| --- | --- | --- | --- | --- | --- | --- |
| *Vibrio* | 36.04444 | 0.6981784 | 0.4314547 | 0.2273886 | 0.5593965 | 0.1931283 |
| *Leuconostoc* | 34.419957 | 73.491717 | 74.485548 | 81.813867 | 86.336943 | 89.993591 |
| *Pantoea* | 21.87304 | 15.783632 | 10.211314 | 6.0913238 | 2.9025565 | 3.0436689 |
| *Cronobacter* | 3.5043395 | 0.0502448 | 0.0421574 | 0.0255015 | 0.0447224 | 0.0202405 |
| *Weissella* | 0.9069841 | 3.4803933 | 7.912418 | 7.2006404 | 5.4583312 | 3.7816047 |
| *Pseudomonas* | 0.6784205 | 0.0284971 | 0.0085632 | 0.0028335 | 0.0043989 | 0.0025301 |
| *Methylobacterium* | 0.5121098 | 0 | 0.0026348 | 0.0021251 | 0.0043989 | 0 |
| *Enterobacter* | 0.3462535 | 3.4586455 | 1.9576845 | 1.2793268 | 0.7177577 | 0.6266129 |
| *Unidentified* | 0.3235334 | 0.5939391 | 0.5256501 | 0.2514734 | 0.3460487 | 0.3179449 |
| *Lactococcus* | 0.2340164 | 1.3378629 | 2.9569467 | 2.2186331 | 1.990513 | 1.1157589 |
| *Enterococcus* | 0.0954242 | 0.0209978 | 0.0197613 | 0.0141675 | 0.0087978 | 0 |
| *Escherichia* | 0.0758849 | 0.6216863 | 0.621163 | 0.5426159 | 0.2558707 | 0.2243325 |
| *Nocardioides* | 0.0495297 | 0.0014998 | 0.0144916 | 0 | 0.0051321 | 0 |
| *Hyphomicrobium* | 0.0477121 | 0 | 0.004611 | 0 | 0 | 0.0050601 |
| *Rosenbergiella* | 0.0358977 | 0.0052495 | 0 | 0 | 0.0014663 | 0 |
| *Streptomyces* | 0.0299905 | 0 | 0.0019761 | 0 | 0.0102642 | 0.0042168 |
| *JADGCD01* | 0.0268097 | 0 | 0 | 0 | 0 | 0 |
| *Neobacillus* | 0.0236288 | 0 | 0.0131742 | 0 | 0 | 0 |
| *Sphingomonas* | 0.0231744 | 0 | 0 | 0.0014168 | 0 | 0 |
| *Staphylococcus* | 0.0213568 | 0 | 0 | 0.0021251 | 0 | 0 |
| *Kosakonia* | 0.0213568 | 0.09749 | 0.2239612 | 0.1097983 | 0.0894448 | 0.0556614 |
| *Sphingobacterium* | 0.0209024 | 0 | 0 | 0.0028335 | 0 | 0.0016867 |
| *Blautia* | 0.0199936 | 0.0067493 | 0.0039523 | 0 | 0.0051321 | 0 |
| *Agrobacterium* | 0.018176 | 0.0037496 | 0 | 0 | 0 | 0 |
| *Lacticaseibacillus* | 0.018176 | 0 | 0 | 0 | 0 | 0 |
| *Mycobacterium* | 0.0172672 | 0 | 0.0032935 | 0 | 0 | 0 |
| *Z2-YC6860* | 0.0172672 | 0 | 0.0065871 | 0 | 0.0249272 | 0.0084336 |
| *Mixta* | 0.0168128 | 0.0892409 | 0.0968303 | 0.0332937 | 0.0293262 | 0.0244573 |
| *Bradyrhizobium* | 0.0168128 | 0 | 0.0032935 | 0.0035419 | 0.0197952 | 0.0219272 |
| *VAZQ01* | 0.0163584 | 0 | 0 | 0 | 0.0021995 | 0 |
| *Arthrobacter* | 0.0154496 | 0 | 0.0059284 | 0 | 0 | 0 |
| *Faecalibacterium* | 0.0131776 | 0.0029997 | 0.0059284 | 0 | 0 | 0.0059035 |
| *Perlucidibaca* | 0.0127232 | 0.0029997 | 0 | 0 | 0 | 0 |
| *Beijerinckia* | 0.0122688 | 0 | 0 | 0 | 0 | 0 |
| *Erwinia* | 0.0104512 | 0.0014998 | 0 | 0 | 0.0014663 | 0 |
| *Solirubrobacter* | 0.0099968 | 0 | 0.0013174 | 0 | 0 | 0 |
| *Ewingella* | 0.0095424 | 0.1147382 | 0.1021 | 0.0913805 | 0.0329919 | 0.0413244 |
| *Bacillus* | 0.0095424 | 0 | 0 | 0.0049586 | 0 | 0 |
| *Actinomycetospora* | 0.0095424 | 0 | 0.0019761 | 0 | 0 | 0 |
| *Sphingomicrobium* | 0.009088 | 0 | 0.0079045 | 0 | 0.0073315 | 0.011807 |
| *Phocaeicola* | 0.009088 | 0 | 0.0065871 | 0 | 0.0029326 | 0.0059035 |
| *Leucobacter* | 0.009088 | 0 | 0 | 0 | 0 | 0 |
| *Bog-532* | 0.009088 | 0 | 0 | 0 | 0.0109973 | 0.0050601 |
| *Curtobacterium* | 0.0086336 | 0 | 0 | 0 | 0 | 0 |
| *Bifidobacterium* | 0.0086336 | 0 | 0.0052697 | 0 | 0 | 0 |
| *Gp1-AA122* | 0.0081792 | 0 | 0 | 0 | 0.0036658 | 0.0042168 |
| *AV40* | 0.0077248 | 0 | 0 | 0 | 0 | 0.0042168 |
| *UBA5189* | 0.0077248 | 0 | 0.0065871 | 0 | 0.0058652 | 0.0025301 |
| *Aureimonas* | 0.0077248 | 0 | 0 | 0 | 0 | 0 |
| *SHVA01* | 0.0072704 | 0 | 0 | 0 | 0 | 0 |
| *PALSA-647* | 0.0072704 | 0 | 0 | 0 | 0.0117305 | 0.0025301 |
| *Hylemonella* | 0.006816 | 0.0014998 | 0 | 0 | 0 | 0 |
| *Nitrospira* | 0.006816 | 0 | 0.0072458 | 0 | 0.0021995 | 0.0059035 |
| *Carnobacterium* | 0.006816 | 0.0442455 | 0.0177852 | 0.0417942 | 0.028593 | 0.0733719 |
| *Roseomonas* | 0.0063616 | 0 | 0 | 0 | 0 | 0 |
| *Lentilactobacillus* | 0.0063616 | 0 | 0 | 0 | 0 | 0 |
| *Micromonospora* | 0.0063616 | 0 | 0 | 0 | 0 | 0 |
| *Conexibacter* | 0.0063616 | 0 | 0 | 0 | 0 | 0 |
| *Priestia* | 0.0059072 | 0 | 0 | 0 | 0 | 0 |
| *WHSN01* | 0.0059072 | 0 | 0.0013174 | 0 | 0.0080647 | 0.0286741 |
| *Collinsella* | 0.0059072 | 0 | 0.0013174 | 0 | 0 | 0 |
| *Ochrobactrum* | 0.0059072 | 0 | 0 | 0 | 0 | 0 |
| *Tumebacillus* | 0.0059072 | 0 | 0 | 0 | 0 | 0 |
| *Rhizobium* | 0.0049984 | 0 | 0 | 0 | 0 | 0 |
| *Gluconobacter* | 0.0049984 | 0 | 0 | 0.0155843 | 0.4259624 | 0.1121662 |
| *Agathobacter* | 0.0049984 | 0 | 0 | 0 | 0 | 0 |
| *Aliidongia* | 0.004544 | 0 | 0.0019761 | 0 | 0.0087978 | 0 |
| *Pedococcus* | 0.004544 | 0 | 0 | 0 | 0 | 0 |
| *SCN-69-37* | 0.004544 | 0 | 0.0013174 | 0 | 0.0014663 | 0 |
| *Corynebacterium* | 0.004544 | 0 | 0 | 0 | 0 | 0 |
| *VXMN01* | 0.0040896 | 0 | 0.0019761 | 0 | 0 | 0.0050601 |
| *VKM-B-2647* | 0.0040896 | 0 | 0 | 0 | 0 | 0 |
| *UBA8199* | 0.0040896 | 0 | 0 | 0 | 0 | 0 |
| *Microvirga* | 0.0040896 | 0 | 0 | 0 | 0 | 0.0016867 |
| *Gp6-AA40* | 0.0040896 | 0 | 0.0085632 | 0 | 0.0051321 | 0 |
| *Falsirhodobacter* | 0.0036352 | 0 | 0 | 0 | 0 | 0 |
| *Achromobacter* | 0.0036352 | 0 | 0 | 0 | 0 | 0 |
| *Acidisphaera* | 0.0036352 | 0 | 0 | 0 | 0 | 0 |
| *Aerococcus* | 0.0036352 | 0 | 0 | 0 | 0 | 0 |
| *JAAYLR01* | 0.0036352 | 0 | 0 | 0 | 0 | 0 |
| *Haematobacter* | 0.0036352 | 0 | 0 | 0 | 0 | 0 |
| *Brachybacterium* | 0.0036352 | 0 | 0 | 0 | 0 | 0 |
| *Azospirillum* | 0.0036352 | 0 | 0 | 0 | 0 | 0.0016867 |
| *AC-14* | 0.0031808 | 0 | 0 | 0 | 0 | 0 |
| *Bosea* | 0.0031808 | 0 | 0 | 0 | 0 | 0 |
| *Actinopolymorpha* | 0.0031808 | 0 | 0 | 0 | 0 | 0 |
| *REEB421* | 0.0031808 | 0 | 0.0019761 | 0 | 0.0036658 | 0 |
| *Ramlibacter* | 0.0031808 | 0 | 0 | 0 | 0 | 0 |
| *SHVJ01* | 0.0031808 | 0 | 0.0019761 | 0 | 0.0021995 | 0.0050601 |
| *Neorhizobium* | 0.0031808 | 0 | 0 | 0 | 0 | 0 |
| *Ammoniphilus* | 0.0031808 | 0 | 0 | 0 | 0 | 0 |
| *Paraperlucidibaca* | 0.0031808 | 0 | 0 | 0 | 0 | 0 |
| *Paenibacillus* | 0.0031808 | 0 | 0.0026348 | 0 | 0.0014663 | 0 |
| *Aquisphaera* | 0.0027264 | 0 | 0 | 0.0014168 | 0.0021995 | 0 |
| *Humisphaera* | 0.0027264 | 0 | 0 | 0 | 0 | 0.0016867 |
| *2-12-FULL-64-23* | 0.0027264 | 0 | 0 | 0 | 0 | 0 |
| *JACCTI01* | 0.0027264 | 0 | 0 | 0 | 0 | 0 |
| *JACQFH01* | 0.0027264 | 0 | 0 | 0 | 0.0014663 | 0.0016867 |
| *Floricoccus* | 0.0027264 | 0 | 0 | 0 | 0 | 0 |
| *Kribbella* | 0.0027264 | 0 | 0 | 0 | 0 | 0.0016867 |
| *Fimbriiglobus* | 0.0027264 | 0 | 0 | 0 | 0 | 0.0016867 |
| *Labrys* | 0.0027264 | 0 | 0 | 0 | 0 | 0 |
| *VFZY01* | 0.0027264 | 0 | 0 | 0 | 0 | 0 |
| *Rhizorhabdus* | 0.0027264 | 0 | 0 | 0 | 0 | 0 |
| *Terriglobus* | 0.002272 | 0 | 0 | 0 | 0 | 0 |
| *Clostridium AM* | 0.002272 | 0 | 0 | 0 | 0 | 0 |
| *Gp1-AA17* | 0.002272 | 0 | 0 | 0 | 0.0080647 | 0.0075902 |
| *Bacteroides* | 0.002272 | 0.0014998 | 0 | 0 | 0 | 0 |
| *Rhodopila* | 0.002272 | 0 | 0 | 0 | 0 | 0 |
| *Anaerostipes* | 0.002272 | 0 | 0 | 0 | 0 | 0 |
| *Lactobacillus* | 0.002272 | 0 | 0 | 0 | 0 | 0.0168671 |
| *MnB-11* | 0.002272 | 0 | 0 | 0 | 0 | 0 |
| *VBCG01* | 0.002272 | 0 | 0.004611 | 0.0014168 | 0 | 0 |
| *JADJLO01* | 0.002272 | 0 | 0 | 0 | 0 | 0 |
| *Bin125* | 0.002272 | 0 | 0 | 0 | 0 | 0 |
| *SCGC-AG-212-J23* | 0.0018176 | 0 | 0.0026348 | 0 | 0 | 0.0025301 |
| *Usitatibacter* | 0.0018176 | 0 | 0 | 0 | 0 | 0 |
| *Saccharopolyspora* | 0.0018176 | 0 | 0 | 0 | 0 | 0 |
| *Pseudonocardia* | 0.0018176 | 0 | 0 | 0 | 0.0021995 | 0.0050601 |
| *Rahnella* | 0.0018176 | 0.0337465 | 0.0105394 | 0.0063754 | 0.0051321 | 0 |
| *Kutzneria* | 0.0018176 | 0 | 0 | 0 | 0 | 0 |
| *Nevskia* | 0.0018176 | 0.0014998 | 0 | 0 | 0 | 0 |
| *Ktedonobacter* | 0.0018176 | 0 | 0 | 0 | 0.0161294 | 0.0033734 |
| *Gemmatirosa* | 0.0018176 | 0 | 0 | 0 | 0 | 0.0025301 |
| *AC-51* | 0.0018176 | 0 | 0 | 0 | 0 | 0.0025301 |
| *CF-113* | 0.0018176 | 0 | 0 | 0 | 0.0102642 | 0.0016867 |
| *Bog-159* | 0.0018176 | 0 | 0 | 0 | 0 | 0.0033734 |
| *Acidisoma* | 0.0018176 | 0 | 0 | 0 | 0 | 0 |
| *CAIXRL01* | 0.0018176 | 0 | 0 | 0 | 0 | 0 |
| *DSYW01* | 0.0018176 | 0.0022498 | 0 | 0 | 0.0029326 | 0.0042168 |
| *13-2-20CM-66-19* | 0.0018176 | 0 | 0 | 0 | 0.0036658 | 0.0050601 |
| *WHTK01* | 0.0013632 | 0 | 0 | 0 | 0 | 0 |
| *Prevotella* | 0.0013632 | 0 | 0 | 0 | 0 | 0 |
| *WHTJ01* | 0.0013632 | 0 | 0 | 0 | 0 | 0 |
| *Methyloceanibacter* | 0.0013632 | 0 | 0.0085632 | 0 | 0 | 0 |
| *Phenylobacterium* | 0.0013632 | 0 | 0 | 0 | 0.0021995 | 0 |
| *JJ008* | 0.0013632 | 0 | 0 | 0 | 0 | 0 |
| *UBA5216* | 0.0013632 | 0 | 0 | 0 | 0 | 0 |
| *Kineococcus* | 0.0013632 | 0 | 0 | 0 | 0 | 0 |
| *UBA4720* | 0.0013632 | 0 | 0 | 0.0014168 | 0.0058652 | 0 |
| *UBA4093* | 0.0013632 | 0 | 0 | 0 | 0 | 0 |
| *Luteitalea* | 0.0013632 | 0 | 0 | 0 | 0 | 0.0050601 |
| *Saccharomonospora* | 0.0013632 | 0 | 0 | 0 | 0 | 0 |
| *Palsa-739* | 0.0013632 | 0 | 0 | 0 | 0 | 0 |
| *Ligilactobacillus* | 0.0013632 | 0 | 0 | 0 | 0 | 0 |
| *Dormibacter* | 0.0013632 | 0 | 0 | 0 | 0.0021995 | 0 |
| *Rubrimentiphilum* | 0.0013632 | 0 | 0 | 0 | 0 | 0.0016867 |
| *Pirellula* | 0.0013632 | 0 | 0.0013174 | 0 | 0 | 0 |
| *Microbispora* | 0.0013632 | 0 | 0 | 0 | 0 | 0 |
| *JAAFHV01* | 0.0013632 | 0 | 0 | 0 | 0.0036658 | 0 |
| *Proteiniphilum* | 0.0013632 | 0 | 0 | 0 | 0 | 0 |
| *JACDCA01* | 0.0013632 | 0 | 0.0098806 | 0 | 0 | 0 |
| *VFJN01* | 0.0013632 | 0 | 0 | 0 | 0 | 0 |
| *DSJA01* | 0.0013632 | 0 | 0 | 0 | 0 | 0 |
| *Cohnella* | 0.0013632 | 0 | 0 | 0 | 0 | 0 |
| *Cytobacillus* | 0.0013632 | 0 | 0 | 0 | 0 | 0 |
| *Rhizomicrobium* | 0.0013632 | 0 | 0.0019761 | 0 | 0.0065984 | 0.0050601 |
| *Blastococcus* | 0.0013632 | 0 | 0 | 0.0014168 | 0.0065984 | 0 |
| *Sinomonas* | 0.0013632 | 0 | 0 | 0 | 0 | 0 |
| *Paracraurococcus* | 0.0009088 | 0 | 0 | 0 | 0 | 0 |
| *Coleofasciculus* | 0.0009088 | 0 | 0 | 0 | 0 | 0 |
| *Cutibacterium* | 0.0009088 | 0 | 0 | 0 | 0 | 0 |
| *Dictyobacter* | 0.0009088 | 0 | 0 | 0 | 0.0021995 | 0 |
| *Ruminiclostridium* | 0.0009088 | 0 | 0 | 0 | 0 | 0 |
| *Aquirickettsiella* | 0.0009088 | 0 | 0 | 0 | 0 | 0 |
| *PALSA-1005* | 0.0009088 | 0 | 0 | 0 | 0 | 0.0033734 |
| *OLB13* | 0.0009088 | 0 | 0 | 0 | 0 | 0 |
| *Clostridium* | 0.0009088 | 0 | 0.0052697 | 0 | 0 | 0 |
| *Streptococcus* | 0.0009088 | 0 | 0 | 0 | 0 | 0 |
| *Stenotrophomonas* | 0.0009088 | 0 | 0 | 0 | 0 | 0 |
| *SIBE01* | 0.0009088 | 0 | 0 | 0 | 0 | 0 |
| *PCC7113* | 0.0009088 | 0 | 0 | 0 | 0 | 0 |
| *Latilactobacillus* | 0.0009088 | 0 | 0 | 0 | 0 | 0 |
| *CAIQIQ01* | 0.0009088 | 0 | 0 | 0 | 0.0021995 | 0 |
| *Endozoicomonas* | 0.0009088 | 0 | 0 | 0 | 0 | 0 |
| *JADJXM01* | 0.0009088 | 0 | 0 | 0 | 0 | 0 |
| *Gp13-AA74* | 0.0009088 | 0 | 0 | 0 | 0 | 0 |
| *R267* | 0.0009088 | 0 | 0 | 0 | 0 | 0 |
| *Gemmata* | 0.0009088 | 0 | 0 | 0 | 0.0014663 | 0.0033734 |
| *40CM-4-65-16* | 0.0009088 | 0 | 0 | 0 | 0.0014663 | 0 |
| *GCA-016699535* | 0.0009088 | 0 | 0 | 0 | 0 | 0 |
| *AG11* | 0.0009088 | 0 | 0.0019761 | 0 | 0.0021995 | 0.0025301 |
| *Rhabdothermincola* | 0.0009088 | 0 | 0 | 0 | 0 | 0 |
| *Acinetobacter* | 0.0009088 | 0 | 0 | 0 | 0.0029326 | 0.0033734 |
| *Patulibacter* | 0.0009088 | 0 | 0 | 0 | 0 | 0 |
| *Flavobacterium* | 0.0009088 | 0 | 0 | 0 | 0.0043989 | 0 |
| *Udaeobacter* | 0.0009088 | 0 | 0 | 0 | 0.0021995 | 0 |
| *UBA12499* | 0.0009088 | 0 | 0 | 0 | 0.0029326 | 0 |
| *Truepera* | 0.0009088 | 0 | 0.0013174 | 0 | 0 | 0 |
| *Phascolarctobacterium* | 0.0009088 | 0 | 0.0013174 | 0 | 0 | 0 |
| *Alicyclobacillus* | 0.0009088 | 0 | 0 | 0 | 0 | 0 |
| *Brevibacterium* | 0.0009088 | 0 | 0.0019761 | 0 | 0 | 0 |
| *Reyranella* | 0 | 0 | 0 | 0 | 0 | 0.0025301 |
| *Rudaea* | 0 | 0 | 0 | 0 | 0.0021995 | 0.0025301 |
| *CAIMXF01* | 0 | 0 | 0 | 0 | 0.0029326 | 0 |
| *Bog-375* | 0 | 0 | 0 | 0 | 0.0073315 | 0 |
| *CAG-452* | 0 | 0 | 0.0032935 | 0 | 0 | 0 |
| *Ruminococcus* | 0 | 0 | 0 | 0.0014168 | 0 | 0 |
| *Bog-209* | 0 | 0 | 0 | 0 | 0.0051321 | 0.011807 |
| *Binatus* | 0 | 0 | 0 | 0 | 0.0014663 | 0 |
| *Rhodococcus* | 0 | 0 | 0.0019761 | 0 | 0 | 0 |
| *SDU3-3* | 0 | 0 | 0.004611 | 0 | 0 | 0 |
| *CAG-177* | 0 | 0 | 0.0019761 | 0 | 0 | 0 |
| *CADCWI01* | 0 | 0 | 0.0013174 | 0 | 0 | 0.0016867 |
| *SCUD01* | 0 | 0 | 0.0026348 | 0 | 0 | 0 |
| *Runella* | 0 | 0 | 0 | 0 | 0 | 0.0016867 |
| *CADEDH01* | 0 | 0 | 0 | 0 | 0 | 0.0025301 |
| *SMWR01* | 0 | 0 | 0 | 0 | 0.0014663 | 0 |
| *Geodermatophilus* | 0 | 0 | 0 | 0 | 0.0043989 | 0 |
| *Bin16* | 0 | 0 | 0.0019761 | 0 | 0 | 0 |
| *Urbifossiella* | 0 | 0 | 0 | 0 | 0 | 0.0025301 |
| *Agromyces* | 0 | 0 | 0.0019761 | 0 | 0 | 0 |
| *UBA6082* | 0 | 0 | 0 | 0 | 0.009531 | 0 |
| *UBA6659* | 0 | 0 | 0.0026348 | 0 | 0 | 0 |
| *UBA668* | 0 | 0 | 0 | 0 | 0.0021995 | 0 |
| *Agriterribacter* | 0 | 0 | 0.0013174 | 0 | 0 | 0 |
| *UBA964* | 0 | 0 | 0.004611 | 0 | 0 | 0 |
| *UBA9968* | 0 | 0 | 0 | 0 | 0.0021995 | 0 |
| *Acidoferrum* | 0 | 0 | 0 | 0 | 0.0124636 | 0.0016867 |
| *UBA2475* | 0 | 0 | 0 | 0 | 0 | 0.0042168 |
| *Acetobacter* | 0 | 0 | 0 | 0 | 0.0256604 | 0 |
| *VGTJ01* | 0 | 0 | 0.0013174 | 0 | 0 | 0 |
| *VGYC01* | 0 | 0 | 0 | 0 | 0 | 0.0050601 |
| *AC-64* | 0 | 0 | 0 | 0 | 0.0021995 | 0 |
| *Virgisporangium* | 0 | 0 | 0 | 0 | 0 | 0.0016867 |
| *WYBL01* | 0 | 0 | 0.0019761 | 0 | 0 | 0.0025301 |
| *2-02-FULL-39-32* | 0 | 0 | 0 | 0 | 0 | 0.0016867 |
| *UBA5150* | 0 | 0 | 0.0013174 | 0 | 0 | 0 |
| *UBA1328* | 0 | 0 | 0.0019761 | 0 | 0 | 0 |
| *Shinella* | 0 | 0 | 0.0039523 | 0 | 0 | 0 |
| *Arboricoccus* | 0 | 0 | 0.0013174 | 0 | 0 | 0 |
| *Bhargavaea* | 0 | 0 | 0.0032935 | 0 | 0 | 0 |
| *Solitalea* | 0 | 0 | 0.0013174 | 0 | 0 | 0 |
| *Sphaerobacter* | 0 | 0 | 0.0019761 | 0 | 0 | 0 |
| *Bariatricus* | 0 | 0 | 0.0019761 | 0 | 0 | 0 |
| *Baekduia* | 0 | 0.0022498 | 0 | 0 | 0 | 0 |
| *BOG-1460* | 0 | 0 | 0 | 0 | 0 | 0.0016867 |
| *Spirillospora* | 0 | 0 | 0 | 0 | 0 | 0.0025301 |
| *REEB76* | 0 | 0 | 0 | 0 | 0.0021995 | 0 |
| *Turicibacter* | 0 | 0 | 0.0013174 | 0 | 0 | 0 |
| *Succinivibrio* | 0 | 0 | 0.0026348 | 0 | 0 | 0 |
| *Sulfotelmatobacter* | 0 | 0 | 0 | 0 | 0.0036658 | 0 |
| *TOLSYN* | 0 | 0 | 0 | 0 | 0.0014663 | 0 |
| *Terracidiphilus* | 0 | 0 | 0 | 0 | 0.0036658 | 0 |
| *Terricaulis* | 0 | 0 | 0.0026348 | 0 | 0 | 0.0016867 |
| *Anaerobutyricum* | 0 | 0 | 0 | 0 | 0.0036658 | 0 |
| *Thermobifida* | 0 | 0 | 0.0026348 | 0 | 0 | 0 |
| *Steroidobacter* | 0 | 0 | 0.0032935 | 0 | 0 | 0 |
| *Pediococcus* | 0 | 0 | 0.0026348 | 0 | 0 | 0.0016867 |
| *QHYU01* | 0 | 0 | 0 | 0 | 0 | 0.0050601 |
| *Flavihumibacter* | 0 | 0 | 0.0013174 | 0 | 0 | 0 |
| *JADJOM01* | 0 | 0 | 0.0013174 | 0 | 0.0014663 | 0 |
| *JADJPH01* | 0 | 0 | 0 | 0 | 0 | 0.0025301 |
| *JAHJRN01* | 0 | 0 | 0.0019761 | 0 | 0 | 0 |
| *JC658* | 0 | 0 | 0.0052697 | 0 | 0 | 0 |
| *Kaistia* | 0 | 0 | 0 | 0 | 0 | 0.0016867 |
| *Kitasatospora* | 0 | 0 | 0 | 0 | 0.0109973 | 0 |
| *Klenkia* | 0 | 0.0029997 | 0 | 0 | 0 | 0 |
| *Kocuria* | 0 | 0 | 0 | 0 | 0.0131968 | 0 |
| *Kouleothrix* | 0 | 0 | 0.0013174 | 0 | 0 | 0.0050601 |
| *QHXM01* | 0 | 0 | 0 | 0 | 0 | 0.0109636 |
| *Fimbriimonas* | 0 | 0 | 0 | 0 | 0.0014663 | 0 |
| *12-FULL-67-14b* | 0 | 0 | 0.0019761 | 0 | 0 | 0 |
| *Fibrobacter* | 0 | 0 | 0.0013174 | 0 | 0 | 0 |
| *Ferruginivarius* | 0 | 0 | 0 | 0 | 0.0021995 | 0.0025301 |
| *Lacipirellula* | 0 | 0 | 0.0039523 | 0 | 0 | 0.0025301 |
| *F1-60-MAGs149* | 0 | 0 | 0.0013174 | 0 | 0 | 0 |
| *Lactiplantibacillus* | 0 | 0 | 0 | 0 | 0.0271267 | 0.0075902 |
| *ELB16-189* | 0 | 0 | 0.0013174 | 0 | 0 | 0 |
| *Frankia* | 0 | 0 | 0 | 0 | 0.0051321 | 0 |
| *JACRPT01* | 0 | 0 | 0 | 0 | 0 | 0.0016867 |
| *Frateuria* | 0 | 0 | 0 | 0.0014168 | 0.028593 | 0.0092769 |
| *JACOUT01* | 0 | 0 | 0.0013174 | 0 | 0 | 0 |
| *Gp22-AA3* | 0 | 0 | 0 | 0 | 0.0014663 | 0 |
| *Gemmatimonas* | 0 | 0 | 0.0026348 | 0 | 0 | 0.0016867 |
| *Gp7-AA6* | 0 | 0 | 0 | 0 | 0.0014663 | 0 |
| *Hafnia* | 0 | 0 | 0.0256897 | 0 | 0 | 0 |
| *Haliangium* | 0 | 0 | 0.0013174 | 0 | 0 | 0 |
| *Geminicoccus* | 0 | 0 | 0.0019761 | 0 | 0 | 0 |
| *GWA2-66-18* | 0 | 0 | 0 | 0 | 0.0014663 | 0 |
| *Hypericibacter* | 0 | 0 | 0 | 0 | 0.0058652 | 0 |
| *J124* | 0 | 0 | 0 | 0 | 0 | 0.0016867 |
| *G192* | 0 | 0 | 0.0013174 | 0 | 0 | 0 |
| *JABDFB01* | 0 | 0 | 0.0013174 | 0 | 0 | 0 |
| *JABDJB01* | 0 | 0 | 0.0026348 | 0 | 0 | 0 |
| *JABFXX01* | 0 | 0 | 0 | 0 | 0 | 0.0033734 |
| *Fusicatenibacter* | 0 | 0 | 0.0032935 | 0 | 0 | 0 |
| *JACDDU01* | 0 | 0 | 0.0013174 | 0 | 0 | 0 |
| *JACDEQ01* | 0 | 0 | 0 | 0 | 0 | 0.0033734 |
| *JACOST01* | 0 | 0 | 0 | 0 | 0 | 0.0016867 |
| *Dongia* | 0 | 0 | 0.0026348 | 0 | 0 | 0 |
| *Lichenihabitans* | 0 | 0 | 0 | 0 | 0.0021995 | 0 |
| *Limosilactobacillus* | 0 | 0 | 0 | 0.0014168 | 0 | 0 |
| *Chondromyces* | 0 | 0 | 0 | 0 | 0.0014663 | 0 |
| *PMG-095* | 0 | 0 | 0.004611 | 0 | 0 | 0 |
| *Cellvibrio* | 0 | 0 | 0.0013174 | 0 | 0 | 0 |
| *Palsa-1233* | 0 | 0 | 0.0013174 | 0 | 0 | 0 |
| *Palsa-513* | 0 | 0 | 0 | 0 | 0 | 0.0025301 |
| *Palsa-851* | 0 | 0 | 0 | 0 | 0.0014663 | 0 |
| *Catenibacterium* | 0 | 0 | 0.0039523 | 0 | 0 | 0 |
| *Paraburkholderia* | 0 | 0 | 0 | 0 | 0.0014663 | 0 |
| *Catellatospora* | 0 | 0 | 0 | 0.0021251 | 0 | 0 |
| *Pectobacterium* | 0 | 0.0179982 | 0.0079045 | 0 | 0 | 0 |
| *CF-13* | 0 | 0 | 0 | 0 | 0 | 0.0016867 |
| *Peribacillus* | 0 | 0 | 0.004611 | 0 | 0 | 0 |
| *CAIXZV01* | 0 | 0 | 0 | 0 | 0 | 0.0025301 |
| *Photobacterium* | 0 | 0 | 0 | 0 | 0.2778653 | 0 |
| *CAIXSE01* | 0 | 0 | 0.0019761 | 0 | 0 | 0 |
| *Pseudolabrys* | 0 | 0 | 0 | 0 | 0 | 0.0109636 |
| *CAIWHR01* | 0 | 0 | 0 | 0 | 0 | 0.0033734 |
| *QHVH01* | 0 | 0 | 0 | 0 | 0 | 0.0025301 |
| *PALSA-968* | 0 | 0 | 0 | 0 | 0 | 0.0025301 |
| *Oscillochloris* | 0 | 0 | 0.0026348 | 0 | 0 | 0 |
| *Luteibacter* | 0 | 0 | 0.0019761 | 0 | 0 | 0 |
| *Oryzihumus* | 0 | 0 | 0 | 0 | 0 | 0.0033734 |
| *Lysobacter* | 0 | 0 | 0.0013174 | 0 | 0 | 0 |
| *MB-PLM-2* | 0 | 0 | 0 | 0 | 0.0029326 | 0 |
| *Massilia* | 0 | 0 | 0 | 0 | 0.0117305 | 0.0059035 |
| *Mesorhizobium* | 0 | 0 | 0 | 0 | 0.0036658 | 0.0025301 |
| *Metabacillus* | 0 | 0 | 0.0118568 | 0 | 0 | 0 |
| *Dellaglioa* | 0 | 0 | 0 | 0 | 0.0161294 | 0 |
| *DSWF01* | 0 | 0 | 0.0019761 | 0 | 0 | 0 |
| *Mitsuokella* | 0 | 0 | 0 | 0.0049586 | 0 | 0 |
| *DP-6* | 0 | 0 | 0 | 0 | 0.0014663 | 0 |
| *DP-1* | 0 | 0 | 0.0039523 | 0 | 0 | 0.0042168 |
| *Myxococcus* | 0 | 0 | 0.0039523 | 0 | 0 | 0 |
| *NIC37A-2* | 0 | 0 | 0.0032935 | 0 | 0 | 0 |
| *Nannocystis* | 0 | 0 | 0.0019761 | 0 | 0 | 0 |
| *Nonomuraea* | 0 | 0 | 0.0013174 | 0 | 0 | 0 |
| *OLB15* | 0 | 0 | 0 | 0 | 0.0021995 | 0 |
| *Oceanobacillus* | 0 | 0 | 0.0026348 | 0 | 0 | 0 |
| *Chryseolinea* | 0 | 0 | 0.0065871 | 0 | 0 | 0.0033734 |
| *ZC4RG20* | 0 | 0 | 0.0098806 | 0 | 0 | 0 |
| *Fungi* |  |  |  |  |  |  |
| *Hanseniaspora* | 4.1538881 | 30.082002 | 55.298666 | 67.635439 | 40.860672 | 48.980324 |
| *Galactomyces* | 11.288855 | 22.643995 | 26.530612 | 24.955823 | 54.054752 | 44.820013 |
| *Pichia* | 9.1134733 | 4.8375976 | 11.659297 | 5.9835936 | 4.2171585 | 5.4023799 |
| *Saturnispora* | 0.3762085 | 0.232707 | 0.3956249 | 0.3352153 | 0.3989373 | 0.3332692 |
| *Candida* | 2.6120179 | 2.4538895 | 0.3357824 | 0.2327352 | 0.0874497 | 0.1533893 |
| *Cladosporium* | 10.112798 | 13.022975 | 1.9439286 | 0.2542848 | 0.0928633 | 0.0487086 |
| *Fusarium* | 0.481469 | 2.431727 | 0.1472313 | 0.0340004 | 0.0249856 | 0.0337542 |
| *Clavispora* | 0.5906279 | 0.5306705 | 0.173353 | 0.0565077 | 0.0295663 | 0.0294815 |
| *Cercospora* | 5.1512631 | 1.7348371 | 0.457842 | 0.039747 | 0.0170735 | 0.0158089 |
| *Vishniacozyma* | 15.964497 | 3.1951045 | 0.3960998 | 0.0430991 | 0.008745 | 0.0132453 |
| *Hannaella* | 11.7034 | 3.1384668 | 0.4288706 | 0.0680008 | 0.014575 | 0.0102544 |
| *Meyerozyma* | 0.7647624 | 0.4420202 | 0.0973627 | 0.0186763 | 0.01166 | 0.0098272 |
| *Papiliotrema* | 13.622128 | 5.2931616 | 0.465441 | 0.0320849 | 0.0104107 | 0.0098272 |
| *Strelitziana* | 0.489266 | 0.4063139 | 0.1486562 | 0.0272961 | 0.0012493 | 0.006409 |
| *Rhodotorula* | 2.430736 | 0.8064715 | 0.0736157 | 0.0052677 | 0 | 0.0051272 |
| *Alternaria* | 0.2975881 | 0.2819572 | 0.0664916 | 0 | 0.01166 | 0.0038454 |
| *Phaeosphaeria* | 0.5704855 | 0.077569 | 0.0451193 | 0.011972 | 0.0024986 | 0.0029909 |
| *Aureobasidium* | 1.2390841 | 1.3962422 | 0.0693412 | 0.0201129 | 0.0012493 | 0.0025636 |
| *Mucor* | 0.0084468 | 1.051491 | 0.0622171 | 0 | 0.0020821 | 0.0021363 |
| *Periconia* | 0.2105208 | 0.0541752 | 0.0118735 | 0.0043099 | 0 | 0.0012818 |
| *Wickerhamomyces* | 0.3879041 | 0.3607575 | 0.0460692 | 0.0114931 | 0.0024986 | 0 |
| *Sarocladium* | 2.4385331 | 1.269423 | 0.1505559 | 0.015803 | 0.0020821 | 0 |
| *Zymoseptoria* | 0.5730845 | 0.2290133 | 0.0284964 | 0.0114931 | 0 | 0 |
| *Pseudopithomyces* | 0.2352116 | 0.2881135 | 0.0660166 | 0.0043099 | 0 | 0 |
| *Erythrobasidium* | 0.2592525 | 0.1612943 | 0.0189976 | 0.0019155 | 0 | 0 |
| *Epicoccum* | 0.4749714 | 0.0948066 | 0.0679164 | 0 | 0 | 0 |
| *Filobasidium* | 0.2397599 | 0.067719 | 0.0028496 | 0 | 0 | 0 |
| *Triangularia* | 0.0058478 | 0.6279396 | 0 | 0 | 0 | 0 |
| *Mycosphaerella* | 0.2345618 | 0.0788003 | 0 | 0 | 0 | 0 |
| *Metschnikowia* | 0.2001248 | 0.0492502 | 0 | 0 | 0 | 0 |
| *Malassezia* | 0.231313 | 0 | 0 | 0 | 0 | 0 |
| *Issatchenkia* | 0 | 0 | 0.0489188 | 0.0387892 | 0.034147 | 0.0320451 |
| *Suhomyces* | 0.0955141 | 0.0197001 | 0.047019 | 0.0090987 | 0.0470563 | 0.0076908 |
| *Fusariella* | 0.0103961 | 0 | 0 | 0.0086198 | 0.0054136 | 0.0076908 |
| *Trichosporon* | 0 | 0.0123125 | 0 | 0 | 0 | 0.0076908 |
| *Humicola* | 0.0038985 | 0 | 0 | 0.0033522 | 0.0024986 | 0.0055545 |
| *Chloridium* | 0.0045483 | 0 | 0 | 0 | 0 | 0.0038454 |
| *Helicoma* | 0 | 0 | 0 | 0 | 0 | 0.0038454 |
| *Penicillium* | 0.0097463 | 0.0948066 | 0 | 0 | 0.0037478 | 0.0034181 |
| *Arxiella* | 0.005198 | 0 | 0 | 0.0033522 | 0.0020821 | 0.0034181 |
| *Aspergillus* | 0.0513307 | 0.1268192 | 0.0113986 | 0 | 0 | 0.0029909 |
| *Stemphylium* | 0 | 0.004925 | 0 | 0 | 0 | 0.0029909 |
| *Kodamaea* | 0.0857678 | 0 | 0 | 0.0019155 | 0.0049971 | 0.0025636 |
| *Fusicolla* | 0.0084468 | 0 | 0.0033246 | 0.0023944 | 0 | 0.0025636 |
| *Mortierella* | 0.0129951 | 0.0123125 | 0.0147231 | 0.003831 | 0.0033314 | 0.0021363 |
| *Exophiala* | 0 | 0 | 0 | 0 | 0.0016657 | 0.0021363 |
| *Pseudocercospora* | 0.0090966 | 0.0751065 | 0.0061742 | 0.0100565 | 0 | 0.0021363 |
| *Glycine* | 0 | 0.0184688 | 0 | 0.0009578 | 0 | 0.0021363 |
| *Nectriopsis* | 0 | 0.0172376 | 0 | 0.0143664 | 0.0112435 | 0.0017091 |
| *Gibellulopsis* | 0.0753717 | 0.0073875 | 0.0056993 | 0.003831 | 0.002915 | 0.0017091 |
| *Microdochium* | 0 | 0 | 0 | 0 | 0.0020821 | 0.0017091 |
| *Debaryomyces* | 0.0090966 | 0.0283188 | 0 | 0 | 0 | 0.0017091 |
| *Psathyrella* | 0.0103961 | 0 | 0 | 0 | 0 | 0.0017091 |
| *Paracylindrocarpon* | 0 | 0 | 0 | 0.0028733 | 0 | 0.0012818 |
| *Devriesia* | 0.1331999 | 0.0541752 | 0.0085489 | 0 | 0 | 0.0012818 |
| *Neptunomyces* | 0.0097463 | 0 | 0 | 0 | 0 | 0.0012818 |
| *Coniothyrium* | 0 | 0 | 0 | 0 | 0 | 0.0012818 |
| *Albifimbria* | 0 | 0 | 0 | 0 | 0 | 0.0012818 |
| *Purpureocillium* | 0 | 0 | 0 | 0 | 0 | 0.0012818 |
| *Verticillium* | 0.0747219 | 0.1305129 | 0.0322959 | 0 | 0 | 0.0008545 |
| *Neodevriesia* | 0.1247531 | 0.1108129 | 0.0109236 | 0 | 0 | 0.0008545 |
| *Acrocalymma* | 0.0331375 | 0.0307814 | 0 | 0 | 0 | 0.0008545 |
| *Conocybe* | 0 | 0 | 0 | 0 | 0 | 0.0008545 |
| *Coniochaeta* | 0 | 0 | 0 | 0 | 0 | 0.0008545 |
| *Bipolaris* | 0.0110458 | 0.0689502 | 0.0541431 | 0 | 0.0054136 | 0 |
| *Talaromyces* | 0.1448955 | 0.1046566 | 0.0094988 | 0 | 0.0049971 | 0 |
| *Plectosphaerella* | 0.061077 | 0.1231254 | 0.0170978 | 0.0023944 | 0.0033314 | 0 |
| *Chrysosporium* | 0 | 0 | 0 | 0 | 0.0024986 | 0 |
| *Phallus* | 0 | 0 | 0 | 0 | 0.0024986 | 0 |
| *Saitozyma* | 0 | 0.0332439 | 0 | 0.0067043 | 0.0016657 | 0 |
| *Akanthomyces* | 0.1767335 | 0.0369376 | 0 | 0 | 0.0016657 | 0 |
| *Neomassarina* | 0 | 0 | 0 | 0 | 0.0016657 | 0 |
| *Parasola* | 0 | 0 | 0.0071241 | 0.0028733 | 0.0012493 | 0 |
| *Pseudoramichloridium* | 0 | 0.004925 | 0 | 0 | 0.0008329 | 0 |
| *Solicoccozyma* | 0 | 0 | 0 | 0 | 0.0008329 | 0 |
| *Rhodosporidiobolus* | 0.1455453 | 0.1329754 | 0.0232721 | 0.0100565 | 0 | 0 |
| *Saccharomyces* | 0 | 0 | 0.0204224 | 0.0095776 | 0 | 0 |
| *Colletotrichum* | 0.1715355 | 0.067719 | 0.0090239 | 0.0076621 | 0 | 0 |
| *Neosetophoma* | 0.0311883 | 0.0320126 | 0.0213723 | 0.0043099 | 0 | 0 |
| *Torula* | 0.0188429 | 0.1305129 | 0.0090239 | 0.0043099 | 0 | 0 |
| *Cyphellophora* | 0.0162439 | 0.0233938 | 0.0018998 | 0.003831 | 0 | 0 |
| *Antennariella* | 0 | 0 | 0 | 0.003831 | 0 | 0 |
| *Paraphaeosphaeria* | 0.0604273 | 0 | 0 | 0.0033522 | 0 | 0 |
| *Dichotomopilus* | 0 | 0 | 0 | 0.0033522 | 0 | 0 |
| *Rachicladosporium* | 0.1046107 | 0.0418626 | 0.0194725 | 0.0028733 | 0 | 0 |
| *Neocosmospora* | 0 | 0 | 0 | 0.0028733 | 0 | 0 |
| *Pestalotiopsis* | 0.0116956 | 0 | 0.0061742 | 0.0023944 | 0 | 0 |
| *Barnettozyma* | 0 | 0.0036938 | 0 | 0.0023944 | 0 | 0 |
| *Coniophora* | 0 | 0 | 0 | 0.0023944 | 0 | 0 |
| *Dioszegia* | 0.0272897 | 0.0554064 | 0 | 0.0019155 | 0 | 0 |
| *Ceratobasidium* | 0.0064976 | 0.0086188 | 0 | 0.0019155 | 0 | 0 |
| *Symmetrospora* | 0.1819316 | 0.1108129 | 0.0052243 | 0.0014366 | 0 | 0 |
| *Montagnula* | 0 | 0.0430939 | 0 | 0.0014366 | 0 | 0 |
| *Articulospora* | 0.0194927 | 0.0086188 | 0 | 0.0014366 | 0 | 0 |
| *Sporobolomyces* | 0.0162439 | 0.0086188 | 0 | 0.0014366 | 0 | 0 |
| *Westerdykella* | 0 | 0 | 0 | 0.0014366 | 0 | 0 |
| *Lecythophora* | 0 | 0 | 0 | 0.0014366 | 0 | 0 |
| *Diaporthe* | 0 | 0.0566377 | 0 | 0.0009578 | 0 | 0 |
| *Phialophora* | 0 | 0.0283188 | 0 | 0.0009578 | 0 | 0 |
| *Nigrospora* | 0.0279395 | 0.0123125 | 0 | 0.0009578 | 0 | 0 |
| *Myrothecium* | 0 | 0.0061563 | 0 | 0.0009578 | 0 | 0 |
| *Sampaiozyma* | 0 | 0 | 0 | 0.0009578 | 0 | 0 |
| *Phyllozyma* | 0 | 0 | 0 | 0.0009578 | 0 | 0 |
| *Preussia* | 0 | 0 | 0.0731407 | 0 | 0 | 0 |
| *Hyphopichia* | 0.1721853 | 0.0344751 | 0.0289713 | 0 | 0 | 0 |
| *Setophoma* | 0.0779707 | 0.0197001 | 0.0232721 | 0 | 0 | 0 |
| *Coprinellus* | 0.0084468 | 0.0160063 | 0.0185227 | 0 | 0 | 0 |
| *Rinodina* | 0 | 0 | 0.0185227 | 0 | 0 | 0 |
| *Roussoella* | 0 | 0 | 0.0175728 | 0 | 0 | 0 |
| *Chlorophyllum* | 0 | 0 | 0.015673 | 0 | 0 | 0 |
| *Ceramothyrium* | 0 | 0 | 0.0137733 | 0 | 0 | 0 |
| *Neoascochyta* | 0.0038985 | 0 | 0.0132983 | 0 | 0 | 0 |
| *Exobasidium* | 0 | 0 | 0.0123484 | 0 | 0 | 0 |
| *Agaricus* | 0 | 0 | 0.0123484 | 0 | 0 | 0 |
| *Lycoperdon* | 0 | 0 | 0.0118735 | 0 | 0 | 0 |
| *Coprinopsis* | 0 | 0.0135438 | 0.0113986 | 0 | 0 | 0 |
| *Xenomycosphaerella* | 0 | 0 | 0.0099737 | 0 | 0 | 0 |
| *Cordyceps* | 0.0363863 | 0 | 0.0090239 | 0 | 0 | 0 |
| *Arthrophiala* | 0.0110458 | 0 | 0.0085489 | 0 | 0 | 0 |
| *Panaeolus* | 0 | 0 | 0.0085489 | 0 | 0 | 0 |
| *Neoceratosperma* | 0 | 0 | 0.0085489 | 0 | 0 | 0 |
| *Meira* | 0.0441834 | 0.0591002 | 0.008074 | 0 | 0 | 0 |
| *Paraconiothyrium* | 0 | 0.0073875 | 0.008074 | 0 | 0 | 0 |
| *Occultifur* | 0.0259902 | 0.0073875 | 0.007599 | 0 | 0 | 0 |
| *Buckleyzyma* | 0 | 0.0135438 | 0.0071241 | 0 | 0 | 0 |
| *Elsinoe* | 0.0077971 | 0.0209313 | 0.0066492 | 0 | 0 | 0 |
| *Veronaea* | 0 | 0 | 0.0066492 | 0 | 0 | 0 |
| *Euoidium* | 0 | 0 | 0.0066492 | 0 | 0 | 0 |
| *Pseudocoleophoma* | 0 | 0 | 0.0056993 | 0 | 0 | 0 |
| *Xylaria* | 0.0019493 | 0 | 0.0052243 | 0 | 0 | 0 |
| *Trichomerium* | 0.0370361 | 0.0233938 | 0.0047494 | 0 | 0 | 0 |
| *Cumuliphoma* | 0.0168936 | 0 | 0.0047494 | 0 | 0 | 0 |
| *Knufia* | 0.029239 | 0 | 0.0042745 | 0 | 0 | 0 |
| *Leptospora* | 0.0038985 | 0 | 0.0042745 | 0 | 0 | 0 |
| *Euteratosphaeria* | 0 | 0 | 0.0037995 | 0 | 0 | 0 |
| *Ceraceosorus* | 0 | 0 | 0.0028496 | 0 | 0 | 0 |
| *Lectera* | 0.0844682 | 0.0061563 | 0.0023747 | 0 | 0 | 0 |
| *Botryotinia* | 0 | 0 | 0.0023747 | 0 | 0 | 0 |
| *Trichoderma* | 0.0740721 | 0.0430939 | 0.0018998 | 0 | 0 | 0 |
| *Sterigmatomyces* | 0 | 0 | 0.0018998 | 0 | 0 | 0 |
| *Xenopenidiella* | 0 | 0 | 0.0018998 | 0 | 0 | 0 |
| *Leptodiscella* | 0 | 0 | 0.0018998 | 0 | 0 | 0 |
| *Pseudophialophora* | 0 | 0 | 0.0014248 | 0 | 0 | 0 |
| *Farysia* | 0 | 0 | 0.0014248 | 0 | 0 | 0 |
| *Dictyosporium* | 0.0701736 | 0 | 0.0009499 | 0 | 0 | 0 |
| *Beauveria* | 0.0129951 | 0 | 0.0009499 | 0 | 0 | 0 |
| *Pyrenochaetopsis* | 0.0747219 | 0.072644 | 0 | 0 | 0 | 0 |
| *Cutaneotrichosporon* | 0.0090966 | 0.0480189 | 0 | 0 | 0 | 0 |
| *Didymella* | 0.0922653 | 0.0394001 | 0 | 0 | 0 | 0 |
| *Teratoramularia* | 0 | 0.0320126 | 0 | 0 | 0 | 0 |
| *Lecanicillium* | 0 | 0.0270876 | 0 | 0 | 0 | 0 |
| *Paratrimmatostroma* | 0 | 0.0258563 | 0 | 0 | 0 | 0 |
| *Chordomyces* | 0.0714731 | 0.0246251 | 0 | 0 | 0 | 0 |
| *Naganishia* | 0.0090966 | 0.0221626 | 0 | 0 | 0 | 0 |
| *Parasarocladium* | 0 | 0.0221626 | 0 | 0 | 0 | 0 |
| *Rhynchogastrema* | 0 | 0.0209313 | 0 | 0 | 0 | 0 |
| *Monocillium* | 0 | 0.0184688 | 0 | 0 | 0 | 0 |
| *Golubevia* | 0.0019493 | 0.0172376 | 0 | 0 | 0 | 0 |
| *Acremonium* | 0.0097463 | 0.0160063 | 0 | 0 | 0 | 0 |
| *Tilachlidium* | 0.0129951 | 0.014775 | 0 | 0 | 0 | 0 |
| *Verrucocladosporium* | 0 | 0.0135438 | 0 | 0 | 0 | 0 |
| *Dendryphion* | 0 | 0.0123125 | 0 | 0 | 0 | 0 |
| *Zopfiella* | 0.0038985 | 0.00985 | 0 | 0 | 0 | 0 |
| *Setophaeosphaeria* | 0 | 0.00985 | 0 | 0 | 0 | 0 |
| *Fitzroyomyces* | 0 | 0.00985 | 0 | 0 | 0 | 0 |
| *Cladophialophora* | 0.0155941 | 0.0061563 | 0 | 0 | 0 | 0 |
| *Carcinomyces* | 0 | 0.0061563 | 0 | 0 | 0 | 0 |
| *Taphrina* | 0 | 0.0061563 | 0 | 0 | 0 | 0 |
| *Microstroma* | 0 | 0.0061563 | 0 | 0 | 0 | 0 |
| *Peniophora* | 0 | 0.004925 | 0 | 0 | 0 | 0 |
| *Sistotrema* | 0 | 0.0036938 | 0 | 0 | 0 | 0 |
| *Curvularia* | 0 | 0.0036938 | 0 | 0 | 0 | 0 |
| *Trichocladium* | 0 | 0.0036938 | 0 | 0 | 0 | 0 |
| *Hansfordia* | 0 | 0.0036938 | 0 | 0 | 0 | 0 |
| *Neurospora* | 0 | 0.0036938 | 0 | 0 | 0 | 0 |
| *Pseudozyma* | 0 | 0.0036938 | 0 | 0 | 0 | 0 |
| *Diutina* | 0.1331999 | 0 | 0 | 0 | 0 | 0 |
| *Gibberella* | 0.0331375 | 0 | 0 | 0 | 0 | 0 |
| *Zasmidium* | 0.0324878 | 0 | 0 | 0 | 0 | 0 |
| *Oidiodendron* | 0.0246907 | 0 | 0 | 0 | 0 | 0 |
| *Bulleromyces* | 0.024041 | 0 | 0 | 0 | 0 | 0 |
| *Zeloasperisporium* | 0.0227414 | 0 | 0 | 0 | 0 | 0 |
| *Podosphaera* | 0.0227414 | 0 | 0 | 0 | 0 | 0 |
| *Spegazzinia* | 0.0220917 | 0 | 0 | 0 | 0 | 0 |
| *Stagonosporopsis* | 0.0207922 | 0 | 0 | 0 | 0 | 0 |
| *Paecilomyces* | 0.0188429 | 0 | 0 | 0 | 0 | 0 |
| *Arthrinium* | 0.0181932 | 0 | 0 | 0 | 0 | 0 |
| *Edenia* | 0.0175434 | 0 | 0 | 0 | 0 | 0 |
| *Cryptococcus* | 0.0155941 | 0 | 0 | 0 | 0 | 0 |
| *Cystobasidium* | 0.0149444 | 0 | 0 | 0 | 0 | 0 |
| *Kockovaella* | 0.0149444 | 0 | 0 | 0 | 0 | 0 |
| *Wallemia* | 0.0136449 | 0 | 0 | 0 | 0 | 0 |
| *Lecidella* | 0.0129951 | 0 | 0 | 0 | 0 | 0 |
| *Mycena* | 0.0116956 | 0 | 0 | 0 | 0 | 0 |
| *Readeriella* | 0.0110458 | 0 | 0 | 0 | 0 | 0 |
| *Ampelomyces* | 0.0084468 | 0 | 0 | 0 | 0 | 0 |
| *Absidia* | 0.0077971 | 0 | 0 | 0 | 0 | 0 |
| *Jaminaea* | 0.0064976 | 0 | 0 | 0 | 0 | 0 |
| *Tetraplosphaeria* | 0.0032488 | 0 | 0 | 0 | 0 | 0 |
| *Kazachstania* | 0.002599 | 0 | 0 | 0 | 0 | 0 |
| *Toxicocladosporium* | 0.002599 | 0 | 0 | 0 | 0 | 0 |
| *Wardomycopsis* | 0.0012995 | 0 | 0 | 0 | 0 | 0 |
